# Rethinking Stress in Parents of Preterm Infants: A Meta-Analysis

**DOI:** 10.1371/journal.pone.0054992

**Published:** 2013-02-06

**Authors:** Renske Schappin, Lex Wijnroks, Monica M. A. T. Uniken Venema, Marian J. Jongmans

**Affiliations:** 1 Department of Medical Psychology and Social Work, Wilhelmina Children’s Hospital, University Medical Center Utrecht, Utrecht, The Netherlands; 2 Department of Special Education, Faculty of Social and Behavioral Sciences, Utrecht University, Utrecht, The Netherlands; 3 Department of Neonatology, Wilhelmina Children’s Hospital, University Medical Center Utrecht, Utrecht, The Netherlands; University of Missouri-Kansas City, United States of America

## Abstract

**Background:**

With improved medical outcome in preterm infants, the psychosocial situation of their families is receiving increasing attention. For parents, the birth of a preterm infant is generally regarded as a stressful experience, and therefore many interventions are based on reducing parental stress. Nevertheless, it remains unclear whether parents of children born preterm experience more stress than parents of term-born children, which would justify these interventions. This meta-analysis provides a comprehensive account of parental stress in parents of preterm infants, from birth of the infant through to their adolescence. Mean levels of stress in specific domains of family functioning were investigated, and stress levels in parents of preterm and term infants, and fathers and mothers of preterm infants, were compared. Furthermore, we investigated moderators of parental stress.

**Methods and Findings:**

A random-effects meta-analysis was conducted including 38 studies describing 3025 parents of preterm (<37 wk) and low birth weight (<2500 g) infants. Parental stress was measured with two parent-reported questionnaires, the Parenting Stress Index and the Parental Stressor Scale: Neonatal Intensive Care Unit. The results indicate that parents of preterm-born children experience only slightly more stress than parents of term-born children, with small effect sizes. Furthermore, mothers have slightly more stress than fathers, but these effect sizes are also small. Parents report more stress for infants with lower gestational ages and lower birth weights. There is a strong effect for infant birth year, with decreasing parental stress from the 1980s onward, probably due to increased quality of care for preterm infants.

**Conclusions:**

Based on our findings we argue that prematurity can best be regarded as one of the possible complications of birth, and not as a source of stress in itself.

## Introduction

Studies regarding preterm birth commonly state that ‘the birth of a preterm infant and subsequent admission to the Neonatal Intensive Care Unit (NICU) are stressful experiences for parents’ [1]–[5]. Indeed, clinical impressions do confirm that preterm birth is a major life-event for parents, and that it is generally accompanied by high levels of parental stress [6]–[9]. Despite these observations, some studies did not find the expected high levels of parental stress [10]–[11]. Therefore, it remains unclear whether parents of preterm-born children experience more stress than parents of term-born children. It is important to investigate this difference and its possible sources, since many interventions for families with preterm-born children are based on reducing parental stress [12]. The current meta-analysis will investigate mean levels of stress in specific domains of family functioning, and compare levels of stress between parents of preterm and term infants, and fathers and mothers of preterm infants. Furthermore, factors related to parental stress, such as birth year or age of the child, will be addressed in moderator analyses.

### Preterm-Term Differences in the First Year of Life

For parents of both preterm, as well as term infants, the birth of a child and the onset of parenthood inevitably cause stress in their life. However, the birth of a preterm infant may be more stressful for parents given the uncertainty about survival of their child, the increased risk of medical complications, and concerns regarding the long term effects of prematurity. Therefore, the differences in stress levels between parents of preterm infants and parents of term infants have often been investigated. Although some studies did find that parents of preterm infants experienced more stress compared to their term counterparts [13], [14], just as many studies proved the contrary [10], [11], [15]–[17]. Studies that reported elevated stress levels in parents of preterm infants unanimously attributed this difference to parental experiences of the preterm birth and subsequent infant illness. However, a methodological explanation for the difference in stress levels is also possible. There could be a selection bias in which only parents who were (still) occupied with the NICU experience participated in these studies and subsequently reported high levels of stress.

Explanations for the lack of difference in stress levels between parents of preterm and term infants during the first year of life are more diverse. One suggestion is that mothers of preterm infants tend to perceive their infants as more healthy than they really are. Another explanation could be, that by rationalizing the emotional impact of preterm birth, mothers are able to maintain control over the situation and diminish parental stress [15]. Furthermore, it could also be that when the infant is older, parents tend to perceive their preterm-born infant as vulnerable, which may lower their expectations of the child’s development. Lowering of expectations may also be prompted by the professional practice to correct physical and cognitive development for prematurity in the first two years of life. As parents have lower expectations, they may experience less stress if their infant lags behind term-born infants [17]. The aforementioned explanations assume that parents have a probably adaptive but distorted view of the true health situation of their infant, resulting in ‘normal’ levels of parental stress. However, it is also possible that due to adequate social support during the neonatal period, stress is not negated, but truly reduced in parents of preterm infants [11]. Furthermore, it could be that birth in itself is such a stressful experience for all parents, that the difference in perceived parental stress between a preterm and a term birth is negligible. This latter explanation is probably mainly dependent on the severity of illness in the preterm infants included in study samples.

Besides theoretical explanations, there are also plausible methodological explanations for not finding a difference in stress between parents of preterm and term infants in the first year of life. It could be that the self-report questionnaires used to measure stress are not sufficient as a measure for parental stress especially during the neonatal period, resulting in unreliable outcomes [11]. For example, the much used Parenting Stress Index contains items like ‘When my child wants something, my child usually keeps trying to get it’ and ‘When playing, my child doesn’t often giggle or laugh’. These items are inappropriate for very young infants, which may lead to missing items on the questionnaire and subsequently distorted outcomes.

### Preterm-Term Differences Beyond the First Year of Life

Looking beyond the first year, the results of studies on stress differences between parents of term-born and preterm-born children are also mixed, with some studies reporting a difference between parents of preterm-born and term-born children [18], [19], and others that found no difference between these groups [20]. It seems reasonable to assume that for relatively healthy preterm-born children, the difference compared with term-born children in terms of health may indeed be negligible at two years of age, which may reduce stress in parents of the preterm-born children. It is also possible that, just as in the neonatal period, parents of preterm-born children may still underestimate the health problems of their child [15]. This underestimation may be due to lack of knowledge or denial of the situation, or both.

However, there are also studies that do report elevated levels of stress in mothers of preterm-born preschoolers compared to mothers of term-born preschoolers [18], [19]. Nonetheless, in these studies, there was only a weak relation between very low birth weight and maternal stress. Furthermore, maternal stress was more related to child behavioral problems, a low level of maternal education and a lack of social support than to biomedical risk factors such as low birth weight. Although a direct link was not established, the effect of preterm birth could be mediated by child behavior problems, since preterm-born children have double the chance of developing these problems [21], [22]. Another probable mediator between preterm birth and parental stress is persisting child health problems [19].

The explanations described above for both similarities and differences in parental stress levels of parents of preterm- and term-born children indicate that when differences are found, there is the established common explanation that preterm birth in itself causes increased levels of parental stress, but when similarities are found, only post-hoc explanations are formulated. Numerous issues may contribute to the different findings, the most important being the selection and characteristics of the sample of infants and their parents, and the instruments that are used to measure stress.

### Mother-Father Differences

Besides differences in stress levels between parents of preterm and term infants, studies also examined differences between fathers and mothers of preterm infants. Most studies report that mothers experience more stress than fathers [23]–[27]. To explain this difference, some authors assume that mothers have higher preliminary expectations of their role as a caregiver due to their bonding with the child during pregnancy [24]. When their preterm infant is admitted to the intensive care unit, mothers may feel as if they are borrowing their baby from the staff, who they regard as the expert caregiver of their infant, and thus lose their role as primary caregiver of the infant, which causes stress. Fathers appear to feel confident in leaving care to the staff, regarding them as expert caregivers [24], [25].

Contradicting the findings above, other studies report that fathers experience more stress compared to mothers [17], or found no differences between mothers and fathers [12]. One explanation for the experience of more stress by fathers is that they may have greater difficulty in understanding their infant’s behavior, which may lead to increased stress [17]. Authors who found similarities between the stress levels of mothers and fathers, argue that parents experience stress from different sources, adding up to the same overall stress level [12]. For example, on the subscale level of their questionnaire, Kaaresen and colleagues found differences between mothers and fathers, with mothers experiencing more stress due to the feeling that they were controlled by their infant’s needs and the lack of emotional support from their spouse, while fathers experienced more stress due to difficulties in understanding their child’s feelings and needs accurately, which consequently may lead to problems in establishing a positive interaction with their infant [12]. Together with the observation that these patterns are identical in parents of term born-infants, this finding may indicate that stress is more likely caused by the traditionally different roles and expectations of mothers and fathers in Western society, and not by prematurity itself.

### Trajectories of Parental Stress

The trajectories of parental stress from birth to school age in parents of preterm-born children have scarcely been studied. The most extensive study is carried out by Singer and colleagues [3], [14], [28], [29], who followed families from the point where the infant reached term age until the age of 14 years. Three groups of infants were investigated: (1) high-risk, very low birth weight (VLBW) infants (infants with chronic lung disorder); (2) low-risk, VLBW infants; and (3) healthy, normal birth weight, term infants. When the infants reached term age, the mothers of the high and low-risk VLBW infants reported more stress compared to the mothers of the term infants. However, for the mothers of the low-risk VLBW infants, stress declined rapidly in the first year until it was the same as the stress experienced by mothers of term infants. By 3 years after their child’s birth, these mothers of low-risk VLBW children actually had the lowest levels of stress. Possibly, these low levels of stress were due to relief after a period of fear for their child’s health. In contrast, mothers of high-risk VLBW children reported an initial decrease in stress from term age until 8 months of age, but subsequently experienced increasing levels of stress up to 3 years of age. When their children were 1 and 3 years of age, these mothers reported more stress than the mothers of low-risk VLBW children and the mothers of term-born children. It was suggested that increasing levels of stress may be due to the opposite effect to that seen in mothers with low-risk VLBW children. It appeared that child development at the age of 2 was predictive of later outcomes, and that these outcomes were negative for many high-risk VLBW children. Singer and colleagues suggested that this resulted in mothers giving up hope that their child’s development would catch up with term-born children, which in turn caused significant maternal stress [28].

The patterns of stress in the different risk groups did not change from the time the child was two years of age to the time the children reached school age [3]. However, during early adolescence the stress of mothers of term infants increased, thereby narrowing the differences between mothers of term-born children and mothers of high-risk VLBW children [29]. During early adolescence, parenting is usually seen as more challenging since children strive for independence and display more difficult behaviors. However, mothers of low-risk VLBW children retained a lower level of stress than mothers in the other two groups. This is probably due to ongoing relief regarding the positive development of their children, although it could also be caused by a delayed onset of adolescence in the preterm-born children [3].

Another interesting study, but covering a much shorter time-span, is the previously-mentioned study by Lau and Morse that compared scores on stress questionnaires with parents’ biological markers of stress during the infant’s first 4 months of life [30]. Differences were found in questionnaire-reported stress 24 hours after birth between mothers of preterm and term infants, but not between fathers. However, when their children were 1 and 16 weeks of age, uncorrected for prematurity, neither mothers nor fathers of preterm infants differed from their term counterparts. To explain the disappearance of this difference, the authors assumed that the parents of the preterm infants had overcome the initial shock of preterm delivery. Due to the great deal of time the parents had spent in the hospital, they may have been well-prepared for caring for their infant. Parents of term infants on the other hand had less assistance in developing their parenting skills, which could result in more insecure feelings about parenting and therefore more stress.

From the above, it is apparent that both longitudinal studies present different stress patterns, although they do not cover the same time-span. Singer’s study reports a difference between mothers’ level of stress in the first year [3], [14], [28], [29], while in Lau and Morse’s study this difference existed only in the first 24 hours after birth [30]. An explanation for the different findings could be that unknown child, parent and environment-related characteristics act as moderators of the relation between the age of the child and parental stress. These characteristics will be described in detail in the next paragraph.

### Factors Related to Parental Stress

Many factors are related to parental stress in parents of preterm infants, with the most prominent ones being birth weight and gestational age of the infant, and the era in which the infant is born. It has generally been found that the lower the birth weight and gestational age, the more stress parents experience [10], [31]–[35], although the magnitude of this relation differs between studies. Other infant and infant health characteristics that are related to increased parental stress include greater expressions of infant distress and fear, infants being difficult to sooth, infants smiling less [10], and the need for prolonged respiratory support [33]. Furthermore, family factors are also related to parental stress, with low social economic status related to increased parental stress [31], [36], and more social support related to reduced parental stress [14], [16], [37].

Another important factor that can be related to parental stress is the birth year of the child. Medical care has improved radically since the invention of the incubator, with milestones including intravenous nutrition, positive pressure ventilation, and surfactant replacement therapy. These advances have improved survival for infants with increasingly lower gestational ages, and have decreased morbidity for low and very low birth weight infants [38], [39]. Besides medical care, hospital practices regarding parent-child interaction have also changed greatly in the last 50 years. Together, altered medical and hospital practices may influence parental stress in the NICU and thereafter.

The influence of the previously-mentioned general infant, family, and environmental factors on parental stress may be a powerful explanation for the reported differences in parental stress between studies. Most studies do not control for general infant and family variables, which may lead to biased samples. In our meta-analysis, we will investigate the influence of infant, family, and environmental factors on the levels of parental stress, the difference in stress between parents of preterm and term infants, and the difference in stress between mothers and fathers of preterm infants.

### Meta-Analysis

The present meta-analysis aims to integrate the findings of previous studies on stress in parents of preterm-born children from birth up to twelve years of age. Our analysis was motivated by the incongruities in empirical findings regarding the existence and magnitude of the difference in parental stress levels between parents of preterm and term infants, and between mothers and fathers of preterm infants. Meta-analysis was used to integrate these findings and to assess the aggregated magnitude of the difference. Furthermore, we aimed to evaluate the strength and direction of the relation between child age and parental stress. An auxiliary goal, which is only possible in meta-analysis, was to investigate the existence of a cohort effect for parental stress.

We tested the following questions: (a) to what extent do parents of preterm and term infants differ in their stress levels?; (b) is this difference related to the child’s age?; (c) to what extent do mothers and fathers of preterm infants differ in their stress levels?; and (d) which factors (including infant year of birth) are related to levels of stress in parents of preterm infants?

## Methods

### Search Strategy

Initially, we used the term *(preterm OR ‘low birth weight’) AND stress* to search the electronic databases The Campbell Library (0 hits) and The Cochrane Library (0 hits). Because these searches did not produce any articles, we assumed that we were investigating a rather unexplored territory. Therefore, we felt it was safe to further limit the search to include only English-language articles, published in peer-reviewed journals. We then searched the electronic databases CINAHL (309 hits), EMBASE (including MEDLINE; 635 hits), PsychINFO (270 hits), and Web of Science (1980 hits), with the same search term. This is a very broad, but sensitive, search term, deliberately chosen to identify a complete sample of relevant studies.

Next, we manually searched the identified articles. The criteria for inclusion of studies in the meta-analysis were: (a) participants were parents of preterm infants (<37 weeks and/or <2500 g) admitted to the NICU, or no-treatment control-group parents of term infants (>37 weeks and/or >2500 g); (b) information regarding mean levels of stress in parents of preterm-born children was available; and (c) stress was measured with a questionnaire (only one article reported biological markers of stress). Of those articles that met these criteria, we examined the reference sections for other relevant articles that had not yet been retrieved. The total search procedure was finalized on January 1^st^ 2011 and resulted in 105 articles.

The collected articles used a diverse range of questionnaires to measure parental stress. Table 1 presents these questionnaires and their authors, together with the articles in which they were published. From Table 1, it is clear that the Parenting Stress Index (Short Form; PSI-SF) [40]–[43], and the Parental Stressor Scale: Neonatal Intensive Care Unit (PSS-NICU) [44], are the most widely-used instruments to measure stress in parents of preterm-born children. To facilitate the interpretation of analysis outcomes by presenting raw means and differences, we chose to only include studies that used the PSI(-SF) and/or the PSS:NICU to measure parental stress, resulting in 85 out of 105 articles. Official translations of both questionnaires were also included, while versions that were different from the original (e.g. including fewer items, presenting different subscales) were excluded (see next paragraph for details).

**Table 1:** Stress Questionnaires Reported by Studies of Parental Stress in Parents of Preterm-Born Children.

Measure	Author	Content	Internal consistency^a^	Article(s)
Daily Hassles Scale	Kanner [111]	Minor events that occurduring daily living	n.a.	Schraeder [112]; Thompson [36]; Tobey [34]; Treyvaud [113]
Depression Anxiety and Stress Scale (Stress Scale only)	Lovibond [114]	General negative affective syndromes	.90	Davis [115]
Life Experiences Survey	Sarason [116]	Negative life stress	.64	Crnic [16]; Crnic [117]
Neonatal Unit Parental Stress Scale	Reid [118]	Parental stress in the NICU	.92	Reid [118]
Nijmegen Parenting Stress Index^c^ (and Short Version)	De Brock [119]	Parenting stress^c^	.95	Colpin [120]; Van der Pal [5], [121]
Parental Stressor Scale: Infant Hospitalization	Miles [122]	Parental stress in hospital	.87–.90	Mackley [123]
Parental Stressor Scale: Neonatal Intensive Care Unit	Miles [44]	Parental stress in NICU	.89–.94	Carter [23], [51]; Cobiella [15]; Denney [52]; Dudek-Shriber [53]; Foster [6]; Meyer [33]; Miles [56]; Miles [25]; Miles [44]; Seideman [57]; Shields-Poë [58]; Spear [68]; see also Table 3
Parenting Stress Index(and Short Form)	Abidin [40]–[43]	Parenting stress	.95	Als [61]; Badr [45]; Barrera [62]; Bendell [60]; Candelaria [63]; Elgen [64]; Elgen [65]; Erdeve [1]; Farel [54]; Farel [55]; Feldman [66]; Geva [67]; Lee [71]; Miceli [59]; Onufrak [46]; Rimmerman [69]; Saylor [47], [48]; Secco [49]; Tallandini [70]; Zahr [50]; see also Table 2
Perceived Stress Scale	Cohen [124]	Perceived stressfulness of situations	.85	Liu [125]
Questionnaire on Resourcesand Stress	Holroyd [126]	Stress in families of childrenwith disabilities	.96^b^	Beckman [37]
Rapid Stress Assessment	Tarsitani [127]	Responses to stressfulsituations	n.a.	Trombini [4]
Stress Appraisal Measure	Peacock [128]	Primary and secondaryappraisals of stress	.51–.90	Feldman Reichman [129]; Rowe [130]
Stress/Arousal Checklist	Gotts [131]	Stress adjectives and statesof arousal	n.a.	Lau [30]
Self-Report Questionnaire	Harding [132]	Stress in developing countries	n.a.	Khan [133]
Swedish Parenthood Stress Questionnaire^d^	Östberg [98]	Parenting stress	.86	Tommiska [20]

a Cronbach’s alpha.

b KR-20.

c Based on the Parenting Stress Index [40]–[43].

d Based on the Parent Domain, questions regarding the behavior and emotions of the parent, of the Parenting Stress Index [40]–[43].

We approached authors of the selected articles and asked them to provide information on sample characteristics and raw stress scores where necessary. Of the 56 authors contacted, 34 were able to provide data. After correspondence, 34 articles describing 28 studies had to be excluded. In 3 of these studies, the authors indicated that, although not mentioned in the article, all parents had participated in an intervention program aimed at reducing stress [45]–[50]. In another 8 studies, scores of preterm infants were combined with scores of other infants (e.g. the sample consisted of the entire NICU population) and authors were not able to provide scores for only preterm infants [23], [44], [51]–[58]. Furthermore, in 1 study, stress scores were combined with scores on another questionnaire, and raw data on stress scores was not available [59], while in 3 studies, stress scores were obtained with a modified or unofficial version of the stress questionnaires [6], [15], [60]. In 9 other studies, stress scores were obtained but not presented in the article, or not presented in a usable format, and the authors could not provide raw means [25], [33], [61]–[68]. Finally, following correspondence with the author of the PSI(-SF), 4 studies were excluded due to incongruities in the number of items and scoring of the questionnaire [1], [69]–[71].

After exclusion, 51 articles describing 38 studies remained for analysis (see Tables 2 and 3). The studies comprised 2599 mothers and 426 fathers of preterm-born/low birth weight children, and involved 710 mothers and 112 fathers in control groups of term-born/normal birth weight children. There were no differences between included and excluded studies, except for that the included studies were on average published 3 years later (*t*_46_ = −2.02; *P* = .049).

**Table 2:** Characteristics of Studies Reporting the Parenting Stress Index (-Short Form) and Included in the Meta-Analysis.

Study	Questionnaire	Study Type	N parents (at 1^st^ measurement)	Birth Weight,Mean (SD), g	Gestational Age, Mean (SD), wk	Birth Year	Age, mo	Maternal Education,Mean (SD), y	Study Location
Askie (2003) [134]	PSI-SF	Intervention; High oxygen saturation	171 mothers	918 (229)	26.6 (1.7)	1996	12	–	Australia
Badr (2009) [135]	PSI-SF	Descriptive	59 mothers	1714 (1243)	31.2 (3.6)	2000	0, 12, 18	–	USA
Benzies (2004) [136]; Harrison[17]; Magill-Evans [137]	PSI	Descriptive	49 mothers, 49 fathers	2328 (364)	34.1 (1.2)	1991	3, 12, 48	14.0 (2.6)	Canada
			control group: 54mothers, 54 fathers	3587 (528)	39.5 (1.1)			14.8 (2.7)	
Browne (2005) [138]	PSI	Intervention; Parent education	22 mothers	1518 (374)	31 (2.7)	1988	0	-	USA
Doussard-Roosevelt (1997) [31]	PSI	Descriptive	41 mothers	930 (-)	28.0 (-)	1987	36	–	USA
Glazebrook (2007) [2]	PSI-SF	Intervention; Parent education	111 mothers	1216 (358)	29.0 (2.2)	2002	3	–	UK
Greco (2005) [139]	PSI-SF	Descriptive	66 mothers	1467 (-)	30.0 (-)	–	9	–	USA
Grunau (2009) [13]	PSI	Descriptive	116 mothers	1263 (486)	29.1 (2.6)	2001	8, 18	14.9 (2.8)	Canada
			control group: 69 mothers	3535 (489)	40.0 (1.1)			16.9 (2.9)	
Halpern (2001) [10]	PSI-SF	Descriptive	23 mothers	<1500	–	–	9	14.4 (2.9)	USA
			control group: 33 mothers	>2500	≥37.0			15.5 (2.2)	
Kaaresen (2006) [12], [89];Olafsen [11], [140]	PSI	Intervention; Parent education	125 mothers, 51 fathers	1393 (430)	30.0 (3.5)	1999	0, 6, 12, 24	13.5 (3.2)	Norway
			control group: 75mothers, 58 fathers	3619 (490)	39.3 (1.3)			14.9 (2.8)	
			control group: 170 mothers	≥2500	≥37.0				
Miles (2006) [141]	PSI	Intervention; Kangaroo care	22 mothers	1133 (367)	28.0 (2.3)	–	12	12.8 (3.5)	UK
Newnham (2008) [142]	PSI	Intervention; Parent education	33 mothers	1619 (599)	33.7 (0.7)	2001	3, 6	13.8 (2.6)	Australia
Ong (2001) [18], [143]	PSI	Descriptive	116 mothers	1257 (154)	31.8 (2.8)	1989	48	8.5 (3.3)	Malaysia
			control group: 96 mothers	3139 (437)	39.1 (1.5)			9.5 (4.4)	
Robson (1997) [144]	PSI	Descriptive	59 mothers	1757 (516)	32.7 (3.6)	1982	69	12.6 (1.9)	Canada
Singer (1996) [3], [14], [28], [29]	PSI	Descriptive	97 mothers	1061 (239)	28.1 (2.3)	1989	1, 8, 12, 24	13.1 (1.9)	USA
			control group: 103 mothers	3466 (537)	39.9 (1.3)			13.4 (2.4)	
Taylor (2001) [19]	PSI	Descriptive	115 mothers	908 (158)	27.5 (2.1)	1982	131	–	USA
			control group: 49 mothers	3375 (614)	≥37.0				
Thomas (2004) [145]	PSI	Descriptive	29 mothers	2009 (625)	33.1 (2.4)	1992	1	–	USA
Veddovi (2004) [146]	PSI	Descriptive	38 mothers	1757 (387)	31.3 (1.6)	1997	12	–	Australia
Youngblut (1998) [147], [148]	PSI	Descriptive	58 mothers	1444 (527)	30.5 (3.2)	1988	48	–	USA
			control group: 61 mothers	3331 (514)	39.6 (1.6)				

**Table 3:** Characteristics of Studies Reporting the Parental Stressor Scale: Neonatal Intensive Care Unit and Included in the Meta-Analysis.

Study	Study Type	N parents(at 1^st^ measurement)	Birth Weight, M (SD), g	Gestational Age,M (SD), wk	Birth Year	Age, wk	Maternal Education,Mean (SD), y	Study Location
Bell (1997) [149]	Descriptive	46 mothers	1351 (-)	31.1 (-)	1996	31	10.8 (1.4)	USA
D’Souza (2009) [150]	Descriptive	62 mothers and 38 fathers	1721 (349)	31.5 (2.5)	2006	32	–	India
Franck (2003) [151], [152]	Descriptive	62 mothers and 22 fathers	1093 (420)	27.6 (2.3)	–	28	–	UK
Holditch-Davis (2003)[153]	Descriptive	30 mothers	1415 (537)	30.3 (2.7)	1994	36	13.9 (1.9)	USA
Holditch-Davis (2009)[154]	Descriptive	177 mothers	1107 (394)	28.3 (2.9)	2001	34	12.6 (1.8)	USA
Holditch-Davis (2007) [155]; Miles [156]	Descriptive	123 mothers	1222 (433)	29.0 (2.6)	1997	35, 44	13.7 (2.2)	USA
Ichijima (2009) [157]	Descriptive	91 mothers and 60 fathers	1555 (522)	31.1 (2.9)	2007	36	–	Japan and New Zealand
Lau (2007) [158]	Descriptive	120 mothers	955 (235)	27.1 (1.6)	1997	31	–	USA
Melnyk (2001) [159]	Intervention; Parent education	22 mothers	1731 (623)	31.6 (2.3)	–	33, 36	–	USA
Melnyk (2006) [160], [161]	Intervention; Parent education	109 mothers and 68 fathers	1627 (468)	31.4 (2.6)	2001	33	–	USA
Meyer (1994) [162]	Intervention; Parent education	16 mothers	1176 (239)	28.8 (2.1)	1986	32, 37	–	USA
Miles (1991) [78]	Descriptive	79 mothers and 43 fathers	1614 (-)	31.0 (-)		31		
Miles (2006) [141]	Descriptive	26 mothers	1133 (367)	28.0 (2.3)	–	33	–	USA
Preyde (2003) [163]	Intervention; Parent buddy program	28 mothers	897 (213)	26.6 (1.7)	2000	28, 32	–	Canada
Reid (2003) [164]	Descriptive	40 mothers	1674 (312)	31.1 (1.7)	2001	31	–	UK
Roberts (2000) [165]	Intervention; Kangaroo care	14 mothers	1481 (409)	31.2 (2.4)	1997	42	–	Australia
Shaw (2006) [166]	Descriptive	32 mothers and 17 fathers	1811 (987)	31.5 (4.9)	–	35	–	USA
Sisk (2006) [167]	Intervention; Lactation counseling	110 mothers	1143 (207)	28.5 (2.1)	2001	29	13.1 (0.2)	USA
Turan (2008) [9]	Intervention; Nursing program	20 mothers and 19 fathers	1450 (417)	31.1 (2.6)	2004	33	–	Turkey
Van der Pal (2007) [27]	Intervention; Neonatal care	64 mothers and 59 fathers	1185 (341)	28.9 (1.9)	2000	31	–	Netherlands

### Data Coding

Studies were identified and coded by the first author. For reliability, a random selection of 20% of the studies (8 studies) was coded by another researcher in the department. The percentage of initial agreement between these two coders was >90%. Disagreements in coding were resolved by reaching consensus between the coders.

Although the concept of stress is subject of a continuous debate, we chose to simply operationalize it according to the definitions in the questionnaires used in this meta-analysis.

#### Parenting Stress Index

The Parenting Stress Index is a self-report questionnaire that was originally developed to identify specific parental, child, and situational characteristics most commonly associated with dysfunctional parenting [40], [41], [43]. It measures the relative magnitude of stress in the parent-child system by integrating the existing scientific knowledge at the time of development with the clinical practice of diagnosing families under stress, thereby assuming that stressors are additive. Thus, the PSI is not based on one specific theoretical framework. Currently, the PSI is mostly used as a screening instrument for the early identification of parent-child systems which are under stress and at risk of developing dysfunctional parenting behavior.

The PSI consists of 120 items with a 5-point Likert scale from which a Life Stress Scale, a Parent Domain, and a Child Domain can be calculated. The Life Stress Scale, which is optional, provides an assessment of the situational stressors in the previous 12 months that can modify parental stress as measured in the Parent and Child Domains. The Parent Domain contains 7 subscales of which 3 scales are based on self-reported personality and pathology variables: (a) ‘Parental Attachment’ assesses the intrinsic investment in the parental role; (b) ‘Competence’ measures the parents’ sense of competence in the parental role; (c) ‘Depression’ examines the extent to which the parent has no emotional and physical energy left to be emotionally available to the child, as well as parental feelings of guilt. The remaining 4 subscales are based on situational variables: (d) ‘Spouse’ examines the emotional and physical support the parent receives in fulfilling the parental role and determines the level of conflict between parents related to parenting; (e) ‘Isolation’ measures the level of social isolation and the availability of social support for the parental role; (f) ‘Health’ assesses the impact of the parent’s health on his or her functioning as a parent; and (g) ‘Role Restriction’ examines the impact of being a parent on the parent’s personal freedom.

The Child Domain consists of 6 subscales, four of which are related to the child’s temperament as reported by the parent: (a) ‘Adaptability’ assesses how well a child handles change and transitions; (b) ‘Mood’ measures child characteristics like excessive crying and withdrawal which parents usually describe as anger-provoking; (c) ‘Demandingness’ examines the pressure the child places on the parent; and (d) ‘Distractibility/Hyperactivity’ measures child behavior that drains the parents’ energy. The other two subscales in the Child Domain measure parent-reported interactive characteristics of the parent-child dyad: (e) ‘Acceptability’ assesses how closely the child meets the expectations of the parents; and (f) ‘Reinforces Parent’ measures the degree to which interaction between parent and child results in a positive affective response from the parents.

Most studies in the meta-analysis present scores on the Parent Domain and Child Domain, or a sum of the two domain scores (Total Stress score), and these domains and the total score were separately coded. Some studies also provided scores of (several) subscales. Since these subscales measure stand-alone concepts which may or may not differ for different study samples and moderators, we decided to also code them separately. Together with the Life Stress scale of the PSI, this meant that for each measurement moment, 17 PSI variables with their standard deviations could be coded.

To place the aggregated mean scores that were calculated in the meta-analysis in perspective, we will give a short description of the normative PSI data. The current edition of the PSI is based on normative data from 2633 mothers of children aged 1 month to 12 years of age, living in the United States of America. This norm group includes the 534 mothers on whom the norms of the first edition of the PSI were based. Mothers were mainly Caucasian (76%) and married (77%). Normative data are also available for a small sample of 200, mainly Caucasian (95%), fathers and a sample of 223 Hispanic parents (the latter not divided in mothers and fathers). Based on these norm groups, a raw Total Stress score of 260 and above, higher than the 85^th^ percentile, is considered to be indicative of a high level of parental stress. The PSI has alpha reliability coefficients ranging from.70 to.95 for the various subscales, and the test-retest reliability over a three month period was good. The validity of the PSI is good, and has been established in numerous studies on children with developmental problems, behavior problems, disabilities and illnesses, as well as studies of at-risk families, and cross-cultural studies [72]–[76].

#### Parenting Stress Index – Short Form

The short version of the PSI, the PSI-SF [42], [43], is a direct derivative of the full-length PSI. The 36 items of the short form are identical to selected items of the full-length PSI. It was developed for clinicians who requested a valid and short instrument that could assess parental stress. The three subscales of the PSI-SF are: (a) Parental Distress, which assesses the distress a parent experiences due to his or her role as a parent; (b) Parent-Child Dysfunctional Interaction, which measures whether the child meets parental expectations and whether the parent derives satisfaction from interaction with the child; and (c) Difficult Child, which examines the child’s self-regulatory capacity. These subscales and the total score of the PSI-SF are all coded in the meta-analysis, although some studies only report the total score. This means that for each measurement moment, four PSI-SF variables were coded. For the purpose of comparison with aggregated means in the meta-analysis, a description of the norms of the PSI-SF is given in the previous paragraph, since the PSI-SF norms are based on 530 subjects from the full-length PSI norm group. A raw total stress score above 90 (or above the 85^th^ percentile) indicates clinically significant levels of stress.

The PSI-SF and the PSI are closely related, and the validity of the PSI-SF has been established by calculating its correlation with the full-length PSI, which was high. The alpha reliability coefficient of the PSI-SF subscales ranges from.80 to.91, and the test-retest reliability over six months was satisfactory.

#### Parental Stressor Scale: Neonatal Intensive Care Unit

The framework for the PSS:NICU is based on stress theory, especially Magnusson’s theory that stressors are physical and psychosocial demands in the environment that approach or exceed the limits of coping [77]. Although this process is applicable to the immediate environment (microsystem), as well as to the broader social setting (exosystem), the PSS:NICU focuses on the microsystem. It measures parental stress in the NICU relating to the appearance of the premature infant, changes in the parental role due to the infant’s illness, and the dynamics and environment of the NICU.

Two different scoring methods can be applied to the 26 items, with 5-point Likert scales and a ‘not applicable (NA)’ option for each item. Both scoring methods are used equally frequently by the studies in the meta-analysis. The first method (metric 1) is recommended by the questionnaire’s authors when the focus is on the NICU environment, and it measures the level of stress produced when a situation occurs by assigning a score of 0 to the NA option. When the focus is on the parents, the second scoring method (metric 2) is recommended. This method measures the overall stress of the environment by assigning a score of 1 to the NA option.

The first version of the PSS:NICU originally consisted of 4 scales: (a) infant behavior and appearance; (b) parental role alterations; (c) sights and sounds; and (4) staff relationship, in other words, the relationship between parents and staff [56]. Due to the observation that two thirds of the parents did not experience the events described by the items on the staff relationship scale, the scale was later removed from the questionnaire [44]. The PSS:NICU also contains a general stress scale, which asks the parents to rate the total experience of having an infant in the NICU on a 5-point Likert scale. The general stress scale should not be confused with the total stress scale. The authors of the PSS:NICU advise not to calculate a total for the scale because of loss of data, but also because the factor analysis supports the three subscales as three separate dimensions. Nonetheless, there are studies in the meta-analysis that do report a total score, including studies from the authors of the PSS:NICU [78], and we also collected these data. Consequently, together with data for the four scales (including the staff relationship scale that was later removed) and the general stress scale, six PSS:NICU variables were coded for each measurement moment.

Reliability of the PSS:NICU subscales ranges from .73 to .94. The correlation between the two metrics is high: .88 to .96 for the subscales and .93 for the total.

#### Moderators

Potential moderators influencing the mean levels of parental stress were coded. The association between prematurity and parental stress, and the association between parental sex and parental stress were separately analyzed by using the preterm-term or mother-father difference as the meta-analytic effect size.

#### Infant characteristics

Several infant characteristics were examined as potential moderators, namely whether the infant was born preterm or term (control group), birth weight, gestational age, percentage of male infants in the sample, severity of neonatal illness, length of stay in the NICU, and child age at the time of the parental stress measurement. Birth weight was coded as the mean birth weight of the sample in grams, and gestational age was coded as the sample’s mean age in weeks, with one decimal place. Severity of neonatal illness proved to be too difficult to code, since studies reported information on neonatal illness in too many different formats that could not be summarized in a meaningful variable representing neonatal illness severity. For example, some studies reported on scoring systems such as CRIB, SNAP, or SNAP-II, some studies reported on Apgar scores, and other studies reported on percentages of infants with neonatal morbidity such intraventricular hemorrhage or perinatal asphyxia. Therefore, we decided to use infant birth weight and gestational age as proxies for neonatal illness. Length of stay in the NICU was coded in mean days. The infant’s age was coded separately for the PSI(-SF) and the PSS:NICU, since the latter was usually completed during the infant’s admission to the NICU, and the PSI(-SF) tended to be completed after discharge from the NICU, often even several years later. Therefore, for the PSI(-SF), the age is given as the age in whole months corrected for prematurity, and for the PSS:NICU, the age is given in weeks after birth.

#### Parent characteristics

For the parents, five characteristics were coded: whether mother or father completed the questionnaires, the age of the mother at birth, the number of years of education of the mother at birth, ethnicity of the participating parent, and socioeconomic status (SES) of the family. Parental stress scores were coded separately for mothers and fathers, in order to compare the two. The mean age and years of education of the mother at birth in the sample were coded in whole years. Ethnicity of the participating parent was coded twofold, first the exact ethnicity of the parents was coded as a string variable, and, secondly, whether the ethnicity of the parents represented a majority or minority in their residing country was coded. Socioeconomic status was coded as low SES, middle SES, and high SES.

#### Environmental characteristics

Year of birth was coded as the birth year of the oldest included children in the study. This variable represents the medical and social changes in the care for preterm infants and their parents.

#### Study characteristics

For each study, the design and location of the study were coded. Study design was coded in terms of whether it was an interventional or a descriptive study. In case of an intervention study, only the (no treatment) control group was included in the meta-analysis. The location of the study was coded as the continent where the study was conducted: Africa, Asia, Europe, North-America, Oceania, or South-America.

### Data Analysis

The studies included in the meta-analysis reported outcomes in means and standard deviations. We entered these statistics in the Comprehensive Meta-Analysis Version 2 program [79]. For PSI(-SF) and PSS:NICU outcomes, raw mean scores were computed as effect sizes, with separate calculations for the two metrics of the PSS:NICU. However, due to the small sample of studies, PSI(-SF) and PSS:NICU difference effect sizes were calculated as Hedges’ *g*, incorporating studies of both metrics for the PSS:NICU. For both the PSI(-SF) and the PSS:NICU, measurements for the same sample reporting stress levels from different parents and at different time-points were meta-analytically combined into one effect size (when a parent or child age-effect was absent). Combining these dependent measures prevents underestimation of the error of the meta-analytic effect.

Effect sizes and confidence intervals for a random-effects model were computed using the Comprehensive Meta-Analysis program. The random-effects model assumes that there is not one underlying true effect within the population, but rather a distribution of true effects. In meta-analysis based on random effects, the effect size is the estimation of the mean of this distribution. Heterogeneity in this estimation is quantified as *Q*, the sum of the weighted squared deviations of study outcomes from the overall effect size, and its significance. A significant *Q* indicates that the true effects vary, although the test has low power in small samples [80]. To quantify heterogeneity, we used the *I* ^2^ statistic [81], which describes the percentage of total variation across studies that is due to real heterogeneity rather than chance. *I* ^2^ can be interpreted as low, moderate, or high, corresponding to values of 25%, 50%, and 75%, respectively.

Random-effects (method of moments) meta-regression was used to assess the relationship between the moderators and the effect-size. Dichotomous variables were dummy coded, and dichotomies containing fewer than four studies were excluded to avoid outcomes based on chance rather than real differences [82]. Due to the relatively small sample of studies, only models with one predictor were tested. A *Q*-test was performed to estimate the significance of the regression model. A significant *Q*_between_ in a regression with one predictor indicates that the predictor explains a significant amount of the effect-size’s variance. The magnitude of this influence was estimated as the 95% confidence interval, calculated with the point estimate and standard error of the predictor.

#### Publication bias

Publication bias refers to the problem that studies missing from the meta-analysis may be systematically different from the included studies. Due to the few and diverse publications on the subject of this meta-analysis, we expected publication bias to be small. Furthermore, researchers in the area of parental stress found both outcome possibilities, namely the presence of absence of a difference (between parents of preterm and term infants, or between fathers and mothers), to be meaningful. Nonetheless, for each effect size, we assessed the possible presence of publication bias by examining the funnel plot, and by testing for asymmetry of the plot (as an indication of publication bias) using Egger’s linear regression method. This method regresses the standard normal deviate (effect size divided by the standard error) against precision (1 divided by the standard error). If the funnel plot is symmetrical, the regression line has an intercept of 0. However, if the plot is asymmetrical, the intercept will not be at 0 and the deviation of the intercept from zero is an indication of the extent of the asymmetry. A *t*-test of the intercept with a 2-tailed *p*-value <.10 indicates significant asymmetry, which implies publication bias [83].

## Results

### Difference in Stress Level between Parents of Preterm-born and Term-born Children

Our trial was reported according to the PRISMA Statement (Figure S1, Table S1). The first analysis examined the mean stress level of parents of preterm infants, measured with the PSI. Between 5 and 13 studies were included in this analysis, depending on the PSI (sub)scale. The results are presented in Table 4 and Figure S2, together with population norms and the number of items in each scale. Regrettably, the difference between the effect-size and the population norm could not be tested with, for example, a *t*-test, due to the fact that the variance of the effect size consisted of variance within and between the groups. Nonetheless, it appeared that the difference was small on all subscales, with a maximum difference of one-third of the population standard deviation for the effect size of the Health subscale. Furthermore, the effect size of both the Total scale of the PSI and the PSI-SF fell between the 55^th^ and 60^th^ percentile, far below the 85^th^ percentile cutoff that indicates high levels of parental stress (raw scores: 260 and 90, respectively). Therefore, on the PSI(-SF), the scores of parents of preterm infants seem to fall within the normal range.

**Table 4:** Meta-Analytic Results of Parents’ Mean PSI Scores.

					95% CI			
Scale	Items	Population Norm^a^ (SD)	k (n)	M	Lower	Upper	*Q*	*I* ^2^
**Child Domain**	47	99.7 (18.8)	13 (797)	102.32	97.08	107.56	221.98**	94.59
Distractibility/Hyperactivity	9	24.7 (4.8)	9 (674)	24.91	23.67	26.15	102.50**	92.20
Adaptability	11	24.9 (5.7)	9 (674)	25.60	24.29	26.92	86.78**	90.78
Reinforces Parent	6	9.4 (2.9)	9 (689)	10.10	9.13	11.08	152.14**	94.74
Demandingness	9	18.3 (4.6)	9 (689)	18.32	16.66	19.98	196.41**	95.93
Mood	5	9.7 (2.9)	9 (674)	10.35	9.68	11.02	71.21**	88.77
Acceptability	7	12.6 (3.5)	9 (674)	13.17	11.75	14.60	195.42**	95.91
**Parent Domain**	54	123.1 (24.4)	11 (734)	122.08	116.95	127.22	100.82**	90.08
Competence	13	29.1 (6.0)	9 (745)	28.16	25.86	30.47	233.72**	96.58
Isolation	6	12.6 (3.7)	8 (630)	12.23	12.67	13.80	25.56**	72.62
Attachment	7	12.7 (3.2)	9 (730)	12.98	11.86	14.10	179.40**	95.54
Health	5	11.7 (3.4)	8 (615)	12.81	12.34	13.29	21.94**	68.10
Role Restriction	7	18.9 (5.3)	8 (630)	18.44	17.79	19.09	18.84**	62.84
Depression	9	20.3 (5.5)	8 (630)	18.78	16.95	20.61	135.10**	94.82
Spouse	7	16.9 (5.1)	8 (630)	17.20^b^	16.27	18.14	41.04**	82.94
**Total**	101	222.8 (36.6)	9 (602)	226.29	212.75	239.83	178.07**	95.51
**PSI-SF Total**	36	-	5 (423)	71.56	68.31	74.81	10.98**	63.56
Life Stress	19	7.8 (6.2)	5 (415)	6.95	3.39	10.52	145.77**	97.26

a Retrieved from Abidin [43].

b Probable publication bias.

* *P*<.10.

** *P*<.05.

Publication bias was only present for the effect size of the Spouse subscale, as indicated by Egger’s regression intercept. Visual inspection of the funnel plot indicated that studies with low stress scores were underrepresented on this subscale. However, since most studies that reported scores on the Spouse subscale of the PSI, reported scores on all of the subscales, it is unlikely that the lack of studies presenting low scores is due to these studies being withheld from publication. Rather, there seems to be a small-study effect in which the effect size is larger in small studies for reasons unrelated to bias. There was heterogeneity in the effect sizes for all PSI scales and this ranged from moderate to high. This indicated that moderator analyses were appropriate for these effect sizes.

Our second analysis concerned the mean stress level in parents of preterm infants, measured with the PSS:NICU. The results of this analysis are presented in Table 5 and Figure S3, with the mean level of stress displayed separately for scoring methods (metric) 1 and 2. Between 7 and 13 studies were included in the analysis of metric 1, and 3 to 8 studies were included in the analysis of metric 2, depending on the subscale. The General Stress scale was not included in the analyses because there were too few studies reporting this scale. For the PSS:NICU, population norms were not available. Nonetheless, the effect size can be interpreted according to the labels of the five points of the Likert scale. On most scales, the effect size was approximately between 2 and 3, in which 2 stands for ‘a little stressful’ and 3 stands for ‘moderately stressful’.

**Table 5:** Meta-Analytic Results of Parents’ Mean PSS:NICU Scores.^a^

	Metric 1						Metric 2					
			95% CI						95% CI			
Scale	k (n)	M	Lower	Upper	*Q*	*I* ^2^	k (n)	M	Lower	Upper	*Q*	*I* ^2^
Sights and Sounds	11 (637)	2.48^b^	2.23	2.72	133.39**	92.50	8 (513)	2.43	2.28	2.59	22.63**	73.49
Infant Appearance	13 (991)	2.76	2.44	3.09	396.23**	96.97	8 (513)	2.70^b^	2.38	3.01	90.38**	93.36
Parental Role Alteration	12 (975)	3.22	2.82	3.61	506.02**	97.83	8 (513	3.11	2.54	3.67	282.92**	97.88
Staff Communication	7 (567)	1.85	1.40	2.31	213.64**	97.19	3 (179)	1.63	1.50	1.76	0.68	0.00
**Total**	9 (718)	2.68	2.21	3.14	716.90**	98.88	4 (426)	2.42^b^	2.03	2.81	117.34**	97.44

a Population norms not available.

b Probable publication bias.

* *P*<.10.

** *P*<.05.

For metric 1, publication bias was present for the effect size of the Sights and Sounds subscale, and, for metric 2, publication bias was seen for the effect sizes of the Infant Appearance subscale and the Total scale, as indicated by Egger’s regression intercept. Inspection of the funnel plots revealed that for all three scales, studies with low stress scores were underrepresented in our sample. Nonetheless, analogous to the publication bias on the PSI, the bias on the subscales is probably due to a small-study effect since most studies reported scores on all subscales. This was, however, not the case for the publication bias for the effect size of the Total score, since this score was often reported as a single score. Heterogeneity was high for all metric 1 effect sizes. For metric 2, heterogeneity was high, except for the effect size of the Sights and Sounds subscale, for which heterogeneity was moderate. The Staff Communication subscale’s effect size showed no heterogeneity, but this was probably due to the very small number of studies included in the effect size calculation for this scale. Except for this last scale, moderator analyses were performed for these effect sizes.

Next, we investigated the difference in stress level between parents of preterm-born and healthy term-born children. This difference can only be measured using the PSI, since the purpose of the PSS:NICU is to measure stress in the NICU, where no healthy term infants are admitted. The results of this analysis are presented in Table 6 and Figure S4. On all scales, parents of preterm-born children showed more stress compared to parents of term-born children. This difference was significant for 9 of the 17 (sub)scales, with effect sizes ranging from 0.18 to 0.33. The largest effect sizes were found in the Child Domain, on the subscales Distractibility/Hyperactivity, Demandingness, and Acceptability. Although there was a clear trend for parents of preterm-born children to experience more stress compared to parents of term-born children, the effect sizes were negligible to small.

**Table 6:** Meta-Analytic Results of Standardized Differences in PSI Scores Between Parents of Preterm-Born and Term-Born Children.

			95% CI			
Scale	k (n_preterm_-n_term_)	Hedges’ g	Lower	Upper	*Q*	*I* ^2^
**Child Domain**	6 (556–548)	0.31	0.14	0.48	9.68*	48.35
Distractibility/Hyperactivity	6 (556–548)	0.28^a^	0.16	0.40	3.71	0.00
Adaptability	6 (613–536)	0.14	−0.04	0.31	10.69*	53.23
Reinforces Parent	6 (571–548)	0.09	−0.08	0.26	9.97*	49.86
Demandingness	6 (571–548)	0.31	0.15	0.46	8.23	39.21
Mood	6 (556–548)	0.23	0.11	0.35	3.22	0.00
Acceptability	6 (556–548)	0.33	0.19	0.47	6.55	23.69
**Parent Domain**	6 (556–548)	0.19	0.07	0.31	5.79	13.70
Competence	7 (686–597)	0.23	0.05	0.41	15.03**	60.08
Isolation	6 (571–548)	0.06	−0.06	0.18	4.58	0.00
Attachment	7 (671–597)	0.15	−0.02	0.33	14.26**	57.91
Health	6 (556–548)	0.18	0.06	0.30	1.50	0.00
Role Restriction	6 (571–548)	0.10	−0.02	0.22	1.70	0.00
Depression	6 (571–548)	0.07	−0.05	0.19	4.11	0.00
Spouse	6 (571–548)	0.09	−0.03	0.21	0.43	0.00
**Total**	6 (486–477)	0.33	0.17	0.49	7.21	30.66
Life Stress	3 (357–291)	0.10	−0.09	0.28	2.70	26.05

Note: Positive difference: parents of preterm-born children experience more stress compared to parents of term-born children.

a Probable publication bias.

* *P*<.10.

** *P*<.05.

Egger’s regression intercept indicated the existence of publication bias for the effect size of the subscale Distractibility/Hyperactivity, but upon visual inspection the funnel plot seemed symmetrical. As explained in the analyses on effect sizes of the mean, publication bias on this subscale is probably due to a small-study effect. Of the 17 effect sizes, 7 had no heterogeneity at all and 5 had significant levels of heterogeneity. Of these last 5 effect sizes, only 3 had more than small levels of heterogeneity, namely the effect sizes for the subscales Adaptability, Competence and Attachment. Moderator analyses were performed for effect sizes on all 10 scales that showed heterogeneity.

### Difference in Stress Level between Mothers and Fathers of Preterm-born Children

Besides differences between parents of preterm-born and term-born children, we also investigated the differences between mothers and fathers of preterm-born children. For the PSI, the sample size of studies reporting stress scores for both mothers and fathers was too small for meta-analysis. Therefore, the difference in stress could only be investigated using the PSS:NICU, which implied that we only had data for the mother-father difference during the NICU period. Results are presented in Table 7 and Figure S5. For all effect sizes except Staff Communication, mothers experienced more stress than fathers, but differences were only significant for two effect sizes. Interestingly, the effect size of the Staff Communication scale indicated that fathers showed more stress on this subscale, although this difference was not significant. The absolute values of all effect sizes ranged from 0.22 to 0.31, with all effect sizes being negligible to small. Thus, there is a small difference between the stress levels of mothers and fathers of an infant admitted to the NICU.

**Table 7:** Meta-Analytic Results of Standardized Differences in PSS:NICU Scores Between Mothers and Fathers of Preterm-Born Children.

			95% CI			
Scale	k (n_mother_-n_father_)	Hedges’ g^a^	Lower	Upper	*Q*	*I* ^2^
Sights and Sounds	6 (333–215)	0.31	0.14	0.48	3.94	0.00
Infant Appearance	6 (333–215)	0.23	0.05	0.41	5.31	5.80
Parental Role Alteration	6 (333–215)	0.30	−0.19	0.79	34.27**	85.41
Staff Communication	4 (267–166)	−0.20	−0.46	0.07	5.03	40.35
**Total**	5 (317–206)	0.22	−0.002	0.44	5.93	32.49

Note: Positive difference: mothers experience more stress compared to fathers. No publication bias was found.

* *P*<.10.

** *P*<.05.

There was no publication bias for any of the effect sizes, and only the effect size of the subscale Parental Role Alteration had a significant and large degree of heterogeneity. We conducted moderator analyses for all effect sizes, except for the effect size of the subscale Sights and Sounds, which showed no heterogeneity at all.

### Moderators of Stress in Parents of Preterm-born Children

We coded twelve moderators to test their impact on parental stress levels. Only eight of these could be analyzed, the moderating variables ‘ethnicity of the parents’, ‘SES of the family’, and’ location of the study’ lacked sufficient variation for analysis in meta-regression. Most parents in the meta-analysis were from a majority group in their residing country, were from middle SES backgrounds, and lived in North-America. The moderator ‘length of stay in the NICU’ was reported in too many different formats to make a meaningful comparison between studies. For example, often days in intensive and medium care were combined, total stay in the primary hospital was given, or total length of stay in both the primary and the secondary hospital was stated. All other moderator variables are presented in Table 8 and 9 and will be discussed below.

**Table 8:** Moderator Analyses for Meta-Analytic Mean PSI and PSS:NICU Scores.

					95% CI				
Moderator	Range	Scale	k (n)	β	Lower	Upper	*Q_between_*	*P_Q_*	*R^2^*
Birth Weight	1257–2009 g	PSI Life Stress	5 (415)	−0.01	−0.01	−0.00	144.53	<.001	.00
	960–1811 g	PSS:NICU Parental Role Alteration^c^	10 (513)	−0.00	−0.00	0.00	2.72	.099	.22
Gestational Age	29.1–33.7 wk	PSI Life Stress	5 (415)	−1.35	−2.53	−0.17	5.04	.025	.74
% Males	45–61%	PSI Role Restriction	8 (630)	−0.09	−0.20	0.02	2.82	.093	.42
Child Age^a^	1–96 mo	PSI Distractibility/Hyperactivity	25 (674)	−0.02	−0.05	0.00	2.96	.085	.43
	1–96 mo	PSI Acceptability	25 (674)	0.02	0.00	0.05	3.93	.047	.59
	1–96 mo	PSI Health	24 (615)	−0.02	−0.03	−0.00	6.96	.008	.00
Maternal Age	26–31 y	PSI Isolation	7 (521)	−0.34	−0.72	0.04	3.00	.083	.01
	26–31 y	PSI Health	7 (506)	−0.23	−0.49	0.04	2.88	.090	.67
	18–32 y	PSS:NICU Sights and Sounds^c^	10 (513)	−0.04	−0.07	0.00	3.22	.073	.00
	18–32 y	PSS:NICU Parental Role Alteration^c^	10 (513)	−0.11	−0.21	−0.02	5.57	.018	.36
Birth Year	1982–2001	PSI Demandingness	9 (689)	−0.22	−0.43	0.00	3.73	.054	.35
	1982–2001	PSI Mood	9 (674)	−0.08	−0.18	0.01	2.86	.091	.24
	1982–2001	PSI Acceptability	9 (674)	−0.17	−0.37	0.03	2.85	.091	.25
	1982–2001	PSI Competence	9 (745)	−0.29	−0.60	0.01	3.50	.061	.33
	1988–2001	PSI Isolation	8 (630)	−0.11	−0.19	−0.03	6.91	.009	.63
	1988–2001	PSI Role Restriction	8 (630)	−0.10	−0.20	−0.00	4.08	.043	.54
	1988–2001	PSI Depression	8 (630)	−0.28	−0.54	−0.03	4.65	.031	.48
Study Design	Descriptive - Intervention	PSS:NICU Total Score^b^	9 (718)	−0.75	−1.41	−0.09	4.94	.026	.51

a For the moderator Child Age, subgroups are not aggregated within studies and *k* represents the number of subgroups.

b Metric 1.

c Metric 2.

**Table 9:** Moderator Analyses for Meta-Analytic Preterm-Term and Mother-Father Standardized Differences in PSI and PSS:NICU Scores.

						95% CI				
Moderator	Range	Difference	Scale	k (n)	β	Lower	Upper	*Q_between_*	*P_Q_*	*R^2^*
Birth Weight	1061–2328 g	P-T	PSI Demandingness	6 (548)	−0.00	−0.00	0.00	3.63	.057	0.90
	1093–1811 g	M-F	PSS:NICU Total Score	5 (206)	−0.00	−0.00	−0.00	4.97	.026	1.00
Gestational Age	27.6–31.5 wk	M-F	PSS:NICU Total Score	5 (206)	−0.14	−0.26	−0.02	5.32	.021	1.00
Birth Year	1988–2001	P-T	PSI Child Domain	6 (548)	0.03	0.01	0.05	5.76	.016	1.00
	1988–2001	P-T	PSI Parent Domain	6 (548)	0.02	−0.00	0.04	2.75	.097	1.00
	1982–2001	P-T	PSI Adaptability	6 (536)	0.03	0.01	0.05	9.41	.002	1.00
	1988–2001	P-T	PSI Reinforces Parent	6 (548)	0.03	0.00	0.05	5.19	.023	0.90
	1988–2001	P-T	PSI Acceptability	6 (548)	0.02	0.00	0.05	4.15	.042	1.00

Note: P-T = difference in stress level between parents of preterm-born and term-born infants; M-F = difference in stress level between mothers and fathers of preterm-born infants.

#### Birth weight

Birth weight was a significant moderator of the effect sizes of the subscales PSI Life Stress and PSS:NICU Parental Role Alteration, the effect size of the preterm-term difference on PSI Demandingness, and the effect size of the mother-father difference on the PSS:NICU Total scale. These four associations were all negative, indicating that the higher the birth weight, the less stress parents experienced on the subscales PSI Life Stress and PSS:NICU Parental Role Alteration, or the smaller the preterm-term difference on PSI Demandingness and the mother-father difference on the PSS:NICU Total scale. For the effect size of PSI Life Stress, this meant that, with a higher infant birth weight, parents experienced fewer stressful situations. The relation with the effect size of PSS:NICU Parental Role Alteration indicated that the higher the infant’s birth weight, the less likely parents were to experience stress related to a change in their parental role in the NICU period. For the effect size of the preterm-term difference of PSI Demandingness, the difference between parents’ feelings about how the child placed demands on them, decreased as the birth weight of the child increased. The same held for the effect size of the mother-father difference: the higher the birth weight of the infant, the smaller the difference in parental stress between mothers and fathers as measured with the PSS:NICU Total scale.

#### Gestational age

In terms of parental stress measures, gestational age of the infant was a significant moderator for two effect sizes. As expected due to the usually high correlation between birth weight and gestational age, both these associations were negative. Regarding the effect size of PSI Life Stress, a higher gestational age of the child was related to less stressful situations reported by parents. For the effect size of the mother-father difference on the PSS:NICU Total scale, the higher the gestational age of the infant, the smaller the difference in parental stress between mothers and fathers.

#### Percentage of male infants

The percentage of male infants was only a significant moderator for the effect size of PSI Role Restriction. The association was negative, indicating that with more male infants in the sample, the parents experienced their parental role to be less restrictive in terms of their freedom.

#### Child age

For the analyses of the moderating variable child age, the aggregated age groups within studies were divided back into the ages for which stress was reported separately within studies. Contrary to our expectations, child age was only related to a few parental stress effect sizes, indicating that parental stress does not change much as the preterm-born child grows older. The direction of the relation between child age and the effect sizes differed. For the effect size of PSI Distractibility/Hyperactivity, the older the child, the less parents felt that the child displayed behaviors associated with ADHD. Furthermore, for the effect size of PSI Health, the older the child, the healthier the parents felt. On the other hand, in terms of the effect size of PSI Acceptability, there was less agreement between the characteristics of older children and the parents’ expectations for the child.

#### Maternal age

Although the range of mean maternal ages was small, it was a significant moderator of the effect sizes of PSI Isolation, PSI Health, PSS:NICU Sights and Sounds, and PSS:NICU Parental Role Alteration. All four associations were negative, which indicates that older mothers experienced less parental stress. For the effect size of PSI Isolation, older mothers were less socially isolated, and based on the effect size of PSI Health, they also felt healthier. During the NICU period, older mothers experienced less stress from their stay in the NICU environment (effect size of PSS:NICU Sights and Sounds) and they felt less stress related to their changed parental roles (effect size of PSS:NICU Parental Role Alteration).

#### Maternal education

Maternal education seemed to be a significant moderator of six PSI effect sizes. However, this effect was solely dependent on the inclusion of the Malaysian study by Ong [18]. Since this study differentiates itself from the other six studies that reported on maternal education by its far lower mean level of education, higher level of stress, and different ethnicity of the sample, we cautiously interpreted from these analyses that maternal education was not a significant moderator of parental stress in our meta-analysis.

#### Year of birth

Year of birth was significantly related to many effect sizes, namely the effect sizes of the PSI subscales Demandingness, Mood, Acceptability, Competence, Isolation, Role Restriction and Depression, and the effect sizes of the preterm-term difference on the PSI Child Domain and Parent Domain, and the subscales Adaptability, Reinforces Parent, and Acceptability. All associations with the effect sizes of the mean were negative, indicating that for infants born in more recent years there was less parental stress. For the effect sizes of the difference, the more recent the birth year of the infants, the larger the difference in stress levels between parents of preterm-born and term-born children.

The interpretation of the relation of birth year and the effect sizes of the mean, was that for a more recent year of birth, the effect sizes indicated that: (a) for PSI Demandingness, parents were less likely to feel that their child placed numerous demands on them; (b) for PSI Mood, parents indicated that their child had a more adaptive emotional functioning; (c) for PSI Acceptability, the child’s characteristics were more likely to match the parents’ prior expectations; (d) for PSI Competence, parents felt more competent in raising their child; (e) for PSI Isolation, parents were less socially isolated; (f) for PSI Role Restriction, parents were less likely to experience their parental role as restricting their freedom; and (g) for PSI Depression, parents felt less depressed.

For the relation between birth year of the infants and the effect sizes of the difference, the interpretation was that the more recent the birth year, the greater the difference between parents of preterm-born and term-born children. In particular, parents of preterm-born children experienced more stress than parents of term children for the effect sizes of: (a) the PSI Child Domain, stress related to the child’s characteristics; (b) the PSI Parent Domain, parental stress; (c) PSI Adaptability, the parents’ feelings that the child is unable to adjust to changes in the physical or social environment; (d) PSI Reinforces Parent, parents experiencing the parental role as restricting their freedom; and (e) PSI Acceptability, how the child’s characteristics match the parents’ expectations.

#### Study design

Study design was a significant moderator of parental stress on the PSS:NICU Total scale. Stress was higher in the four descriptive studies compared to the five intervention control-group studies. It is likely that the participants in the control groups of the intervention studies were positively influenced by the knowledge that they were participating in an intervention, a well known phenomenon [84].

## Discussion

Almost every publication about preterm birth and parenting refers to preterm birth as a stressful experience for parents, despite the inconsistent results of empirical studies that investigated stress in parents of preterm infants. Therefore, the first objective of our meta-analysis was to aggregate the outcomes of empirical studies to determine the existence and size of the difference in parent-reported stress (by means of questionnaires) between parents of preterm versus parents of term infants. Additionally, we tested whether mothers and fathers of preterm infants reported different levels of stress, and we examined the impact of several moderators on parental stress.

### Parents of Preterm-born and Term-born Children

The main results of our meta-analysis indicate that parents of preterm infants experience only slightly more stress compared to parents of term infants. Clinically, the difference between both groups of parents is negligible. On the PSI, parents of preterm infants scored between the 55^th^ and 60^th^ percentile, with a maximum deviation from the population norm of one-third SD. On the PSS:NICU, parents of preterm infants indicated that they experienced little to moderate levels of stress.

The finding that the stress level in parents of preterm infants is only slightly higher than that in parents of term infants is probably due to the inclusion of relatively healthy preterm infants in the studies analyzed. Inspection of the exclusion criteria of those studies reporting PSI(-SF) scores, revealed that 14 out of 19 studies excluded infants based on health criteria, usually congenital anomalies. Preterm infants with congenital anomalies have high mortality and morbidity and, although a direct link between infant health and parental stress has not been established, it is likely that parents of these infants would experience high levels of stress. However, the impact of excluding infants with congenital anomalies on the stress levels found in our meta-analysis are small, since preterm infants with congenital anomalies make up only 8.7% of the preterm population [85]. Nonetheless, it is remarkable that studies investigating parental stress collectively exclude the most unwell infants with, probably, the most stressed parents. Consequently, instead of being applicable to the entire population, the results only apply to a select group of infants and parents. Therefore, we strongly advocate including preterm infants with congenital anomalies and other medical conditions in forthcoming studies on parental stress.

Putting the omission of infants with congenital anomalies aside, we should also consider the possibility that the finding that parents of preterm infants experience only slightly more stress than parents of term infants is an artifact of the instrument used to measure parental stress. It may be that in the real-world situation, parents of preterm infants do experience markedly more stress than their term counterparts, but that this difference cannot be captured with the PSI.

Although the validity and reliability of the PSI have extensively been established, it remains a subjective instrument that measures parents’ *perceptions* of parental stress. Observations or biological markers of stress may reveal higher levels of stress in parents of preterm infants, although the only study on cortisol and tribulin levels found that parents of preterm infants actually had lower levels of these stress markers than parents of term infants [30]. Another option is in-depth interviews. In a retrospective study, Wijnroks found that 38% of mothers of preterm infants recollected many concerns and considerable feelings of anxiety about their infant’s health during the neonatal period [35].

Nonetheless, the PSI and PSS:NICU remain the most commonly-used instruments to measure parental stress in, and after, the NICU. Furthermore, we did not find high levels of variability in the effect size of the difference between parents of preterm and term infants, indicating that our findings reflect a consistent trend across studies. Therefore, we argue that our findings are not merely an artifact of the measurement instrument, but also need to be explained in terms of infant, parental and environmental variables influencing parental stress.

The notion that parents of preterm infants do not experience markedly more stress than parents of term infants, is counterintuitive. Therefore, studies that reported this outcome put forward post-hoc explanations focusing on maternal coping strategies to reduce stress [15], [17]. Usually, it is suggested that adapted parental expectations about infant outcomes result in lower levels of parental stress. However, this is contradicted by recent findings that mothers of preterm infants had the same expectations for the timing of developmental milestones as mothers of full term infants [86].

A more plausible explanation is that, for the group of parents of preterm infants, stress levels vary based on infant health characteristics. This is supported by our finding that the difference in stress levels between parents of preterm and term infants is influenced by the birth weight of the infant: the higher the birth weight, the smaller the difference. This might be because lower birth weight is synonymous with higher infant morbidity and mortality [21], thus increasing parental stress. However, because the mean stress level in all parents of preterm infants does not exceed that of parents of term infants, there should be at least as many parents without stress as parents with high levels of stress, to even out the differences.

In conclusion, it seems that thus far, the literature on parental stress in both parents of preterm and term infants does not provide adequate explanations for the small but consistent stress differences we found between both groups of parents. Future studies should search for such explanations, preferably using longitudinal designs that start before birth to capture preexisting family circumstances, and should examine the influence of pregnancy, birth, and postnatal variables on these family circumstances.

### Mothers and Fathers

The second important result of our meta-analysis was that mothers experience slightly more stress than fathers in the NICU period. Only two of the five effect sizes regarding PSS:NICU subscales indicated that mothers scored slightly higher than fathers, with negligible to small effect sizes. To date, mothers have been expected to experience markedly more stress. With no structural evidence to support this, we assume that this notion was based on traditional sex roles, thereby negating the importance of fathers. Theoretical models also only concerned mothers, and although the outcome in stress levels of these models may be the same for fathers, it is unclear whether the sources of stress leading to this outcome are equivalent for both parents. Interestingly, in studies of healthy term infants, the role of fathers is usually negated [87]. Some of those studies that did investigate both mothers and fathers, found that fathers reported parental stress related to their marital status, parity of the infant, and temperament of the infant, whilst they did not report stress from delivery-related variables [87]. These reported variables could also play a role in the development of stress in fathers of preterm infants.

Our finding that, as the result of staff communication, fathers experience more stress than mothers (although not significantly more) is an indication of different sources of stress for both parents. Already in the 1960s, the role of the father was solely limited to acquiring information about the infant’s health, while both parents resided at home [88]. However, even in more recent times, fathers have been reported to comply with their traditional role [89], playing a mainly instrumental role and leaving the emotional involvement with neonatal care to the mother. Consequently, the observation that fathers experience more stress from communication with hospital staff and less from the appearance and surroundings of their infant is a logical fulfillment of their instrumental role. However, this does not automatically deny their emotional involvement with their infant. Unfortunately, our data is not sufficiently extensive to unravel whether parents experience stress from different sources and how this is related to traditional parental roles and emotional involvement with their infant.

Although the difference in stress between mothers and fathers is only significant on some scales, we did find that this difference decreases when infant gestational age and birth weight are higher. This is another indication that mothers and fathers differ in the sources that cause stress. Since lower gestational age and lower birth weight are related to greater infant mortality and morbidity, infant health appears to be one of the factors that influences maternal and paternal stress differently.

### Birth Weight and Gestational Age

The birth weight and gestational age of the children were related to life stress, as measured with the PSI. Life stress represents positive and negative stressful circumstances that are beyond the parents’ control and tend to intensify parental stress. Parents with lower birth weight infants and with lower gestational age infants, reported more life stress. Although this relationship does exist, as a whole, parents of preterm infants only experience slightly more stress than parents of term infants on this measure. However, smaller infants are usually more ill and stay in the NICU for longer periods. This often puts a strain on marital relations, and one of the parents may choose to decrease working hours or to cease working altogether, resulting in a lower family income [90], [91]. Both circumstances contribute to the development of life stress and may thereby influence parental stress. In this light, it would have been interesting to investigate the relation between parental stress and small-for-gestational-age (SGA) infants, since these infants suffer from more health problems than those with solely a low birth weight or low gestational age [92], [93]. We believe that parents of these infants would experience more stress. Unfortunately, the percentage of SGA infants was not usually mentioned in the studies included in our meta-analysis, which made it impossible to investigate this moderator.

### Percentage of Male Infants

Parental stress due to parents feeling restricted in their freedom and identity formation was related to the proportion of male infants in study samples. Interestingly, the higher the percentage of boys in the sample, the less stress parents reported. We would expect parents of sons to experience more stress since morbidity and mortality is higher in male infants than female infants [94]. However, parents may not be aware of this difference, and therefore parents of sons may not be more concerned about their infant’s health than parents of daughters.

An overview of differential parenting found that parents tend to treat their sons and daughters similarly on many domains, but that boys seem to have fathers who are more engaged, parents with greater marital stability and happiness, and receive more financial support from their parents [95]. Although the underlying mechanisms of these advantages for boys are not clear, it is plausible that greater marital stability and parental happiness decrease parental stress in families with sons. Further research should examine whether these patterns of differential parental involvement are also apparent in families with preterm-born children.

### Child Age

The influence of the age of the child on parental stress was not as marked as we had expected. Based on the results of longitudinal studies, we had expected child age to have a more overarching effect, influencing almost all stress dimensions. The usual pattern described in studies is that stress is highest in the neonatal period, and then decreases as the child grows older. On two of the three scales on which an effect of age was found, stress did indeed decrease. However, on one scale stress increased with child age. From these results, we conclude that the relation between parental stress and child age may not be as clear-cut as previously believed, but that there are different developmental trajectories for different dimensions of parental stress.

We found that, when the children were between 1 and 96 months of age, parental stress regarding distractibility of their child decreased. An explanation for this decrease could be that in the first years of life, preterm-born children are more irritable and difficult to soothe, and often suffer from problems with self-regulation [96], [97]. As a result of neurological maturation and parental coping, these problems may become less prominent at school age, when, in many preterm-born children, cognitive and behavioral problems become more noticeable [21]. At this time, parental stress about their child’s acceptability increases, indicating that the child may be failing to meet parental expectations regarding physical, intellectual, and emotional capabilities. Both sources of stress, distractibility and cognitive or behavioral problems in the child, may be expressions of the same underlying neurological impairment, with different manifestations at different ages.

Although stress about the child’s acceptability seems to replace stress about distractibility to an equal extent, stress about the parents’ own health only decreases as the child grows older. Since the questionnaires are usually completed by mothers, this could be the result of a complicated pregnancy and postpartum period, after which stress decreases. Another or additive explanation could be that, as the child becomes more independent and in less need of intensive parental care, there is less demand on parents in terms of energy and therefore they feel healthier.

### Maternal Age

Besides the age of the child, the age of the mother also influences some aspects of parental stress. In the mean maternal age range of 26 to 31 years, older mothers felt less isolated and healthier. During the NICU period, they also experienced less stress from the NICU environment and their changed parental role. These results suggest that mothers aged around 30 have a more established social network, and may therefore experience less stress from the admission of their child to the NICU. It could be that around this age, although parents are still young and healthy, they are also settled with a stable relationship, permanent jobs, and their own home. This explanation may also hold true for parents of term infants, as they experience the least social isolation between the ages of 25 and 34 years, compared with younger and older parents [98].

### Maternal Education

Maternal education was the only moderator that did not have a significant effect on any aspect of parental stress. This was unexpected, since maternal education can be seen as a proxy for family socioeconomic situation and is therefore negatively related to parental stress in most studies (refs). Furthermore, low levels of maternal education mediate the effects of inadequate prenatal care and maternal smoking habits, and are therefore a risk factor for preterm birth (ref).é.

### Year of Birth

Birth year of the infants was the most influential moderator among the variables related to parental stress examined in our meta-analysis. On seven domains of parental stress, stress declined over two decades. Furthermore, with more recent birth years there was an increase in the difference between parents of preterm and term infants on five domains of parental stress. Birth year data was available from 1982/88 to 2001, a period during which neonatal care changed markedly with, for example, the use antenatal corticosteroids and surfactant replacement therapy [99]–[101]. Other important changes in hospital practices during this period included the introduction of family-centered care and kangaroo care, in which parents are encouraged to hold their infant skin-to-skin, preferable to their bare chest [102]–[104].

Although it is not possible to pinpoint which specific medical or social change caused parental stress to decrease over the course of 20 years, overall there does appear to have been a positive influence. Over two decades, parents experienced less stress due to the demands placed on them by their child, the affective functioning of their child, and how the child meets their expectations. The improvements in these domains are indicative of improved neurological outcome in preterm-born children [101], [105]. Parents also reported less stress regarding their own competence as a parent, their feeling of restriction of freedom, depressed feelings and social isolation. This could be due to social practices in the NICU, but the higher prevalence of preterm birth could also make it a more familiar, and therefore less stressful, phenomenon [106], [107].

Interestingly, the difference between parents of preterm and term infants increased between the 1980s and 2001. Since we know that, on the whole, stress decreased in parents of preterm infants, this increased difference can only be explained by an even greater decrease in stress in parents of term infants. Unfortunately, as yet, there is no review or meta-analysis on parental stress in parents of healthy term infants. Moreover, only few studies have investigated stress in parents of healthy, term newborns. Most studies focus on stress-related psychiatric disorders such as post-traumatic stress disorder and depression in mothers. Those studies that did investigate parental stress in itself, are usually concerned with specific family situations, for example families with disabled children [108], families with chronically ill children [109], and families with children born after in vitro fertilization [110]. Therefore, there is no empirical evidence for our assumption that stress has decreased more in parents of term infants than in parents of preterm infants during the last 20 years.

### Limitations and Future Directions

We have conducted our meta-analysis with the greatest care, but nonetheless a number of limitations remain. First, we based our meta-analysis on the outcomes of two of the most commonly-used stress questionnaires. Although this enables us to translate meta-analytical results back to the original measure, it also raises the question as to whether these two instruments cover the full scope of parental stress. Furthermore, this increases the chances that findings are merely an artifact of the questionnaire and do not represent the real-world situation. With the same finding on multiple instruments, indications for the true existence of the effect in the population are stronger. An additional problem with the PSI questionnaire is its many subscales, leading to multiple comparisons and therefore an increased likelihood of a type I error, or the rejection of a true null hypothesis.

A second limitation is that due to the limited number of studies included in some analyses, not all moderators could be tested for every stress (sub)scale. Furthermore, some important moderators lacked sufficient variance for analysis with meta-regression. This implies that our results are mainly based on North-American, middle SES, ethnic majority groups. Finally, our choice of moderators was restricted to the variables included in the primary studies. There may be other variables that influence parental stress that are not included in our analyses. For example, we would expect that parity or previous fertility treatment also affects parental stress.

For future studies we have two important directions. First, it would be meaningful not to see preterm birth in itself as a stressor, but to consider prematurity to be one of the complications of birth. It would be particularly interesting to identify which aspects of preterm birth induce stress, in which we assume that unexpected complications may be more stressful than expected complications as these violate parent’s expectations. Knowing the exact sources of stress for individual parents could also help in the early determination of parents who may need psychological assistance. Second, fathers should be explicitly included in studies regarding parental stress. We assume that the experience of (preterm) birth differs for mothers and fathers, and that they partly experience stress from different sources. Furthermore, birth puts a strain on the relationship, and the interplay between (preterm) birth, parental stress, and spousal relations in terms of harmful and protective factors needs to be studied further.

### Conclusion

The results of our meta-analysis indicate that, probably due to increased quality of care for preterm infants, parental stress decreased from the 1980s onward. Furthermore, our results challenge the common notion that, from their child’s birth through to adolescence, parents of preterm-born children experience markedly more parental stress compared to parents of term-born children. This implies that prematurity can best be regarded as one of the possible complications of birth, and not as a source of stress in itself. Furthermore, our results indicate that mothers experience only slightly more stress than fathers, although possibly from different sources. Nonetheless, with fathers having long been neglected in studies on prematurity research, we argue that future research should explicitly include them.

## Supporting Information

Figure S1 **PRISMA Flow Diagram.**
(DOC)

Figure S2 **Forest Plots for Meta-Analytic Results of Parents’ Mean PSI Scores.** A. Child Domain; B. Distractibility/Hyperactivity; C. Adaptability; D. Reinforces Parent; E. Demandingness; F. Mood; G. Acceptability; H. Parent Domain; I. Competence; J. Isolation; K. Attachment; L. Health; M. Role Restriction; N. Depression; O. Spouse; P. PSI Total; Q. PSI-SF Total; R. Life Stress.
(PDF)

Figure S3 **Forest Plots for Meta-Analytic Results of Parents’ Mean PSS:NICU Scores.** A. Sights and Sounds Metric 1; B. Sights and Sounds Metric 2; C. Infant Appearance Metric 1; D. Infant Appearance Metric 2; E. Parental Role Alteration Metric 1; F. Parental Role Alteration Metric 2; G. Staff Communication Metric 1; H. Staff Communication Metric 2; I. PSS:NICU Total Metric 1; J. PSS:NICU Total Metric 2.
(PDF)

Figure S4 **Forest Plots for Meta-Analytic Results of Standardized Differences in PSI Scores Between Parents of Preterm-Born and Term-Born Children.** Note. Favours A = Parents of term-born children experience more stress than parents of preterm-born children; Favours B = Parents of preterm-born children experience more stress than parents of term-born children. A. Child Domain; B. Distractibility/Hyperactivity; C. Adaptability; D. Reinforces Parent; E. Demandingness; F. Mood; G. Acceptability; H. Parent Domain; I. Competence; J. Isolation; K. Attachment; L. Health; M. Role Restriction; N. Depression; O. Spouse; P. PSI Total; Q. Life Stress.
(PDF)

Figure S5 **Forest Plots for Meta-Analytic Results of Standardized Differences in PSS:NICU Scores Between Mothers and Fathers of Preterm-Born Children.** Note. Favours A = Fathers experience more stress than mothers; Favours B = Mothers experience more stress than fathers. A. Sights and Sounds; B. Infant Appearance; C. Parental Role Alteration; D. Staff Communication; E. PSS:NICU Total.
(PDF)

Table S1 **PRISMA Checklist.**
(DOC)
