# Supplementary material for: Rethinking Stress in Parents of Preterm Infants: A Meta-Analysis
Source: PLoS One. 2013 Feb 6;8(2):e54992. doi: 10.1371/journal.pone.0054992 (PMC3566126; doi:10.1371/journal.pone.0054992)
Supplement: Figure S1 — PRISMA Flow Diagram. (DOC) [file pone.0054992.s001.doc]

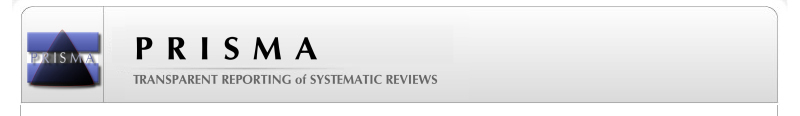
**PRISMA 2009 Flow Diagram**

**Screening**

**Included**

**Eligibility**

**Identification**

Records identified through database searching
(n = 3194 )

Additional records identified through other sources
(n = 0 )

Records after duplicates removed
(n = 3194 )

Records screened
(n = 3194 )

Records excluded
(n = 3089 )

Full-text articles assessed for eligibility
(n = 105 )

Full-text articles excluded, with reasons
(n = 54 )

Reasons:

- Different measurement of stress

- Parents received intervention

- Authors could not provide raw means

- Modified version of questionnaire used

- Improbable scores (outlier)

Studies included in qualitative synthesis
(n = 38 )

Studies included in quantitative synthesis (meta-analysis)
(n = 38 )
