# Supplementary figures and images for: Rethinking Stress in Parents of Preterm Infants: A Meta-Analysis
Source: PLoS One. 2013 Feb 6;8(2):e54992. doi: 10.1371/journal.pone.0054992 (PMC3566126; doi:10.1371/journal.pone.0054992)

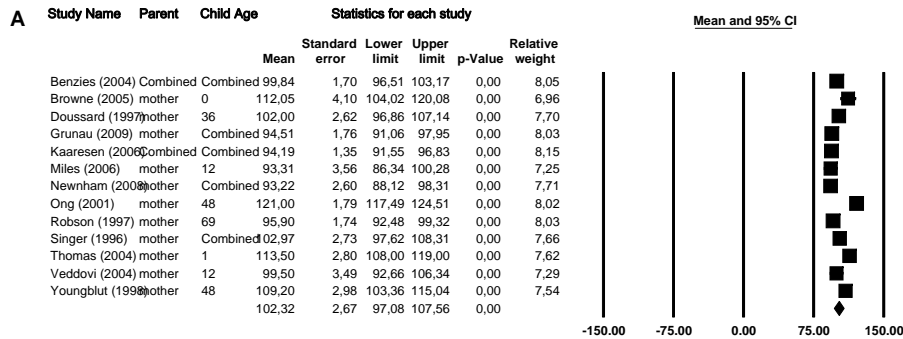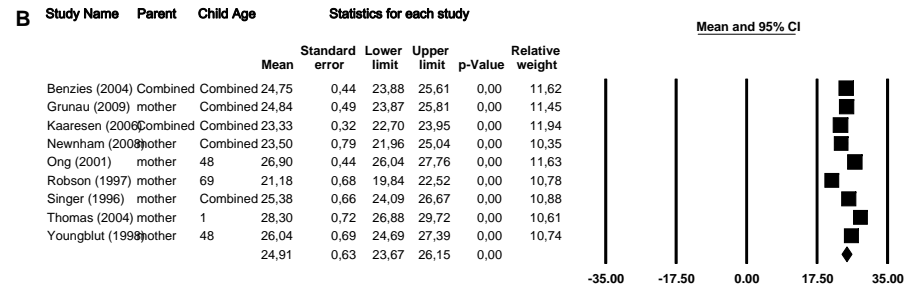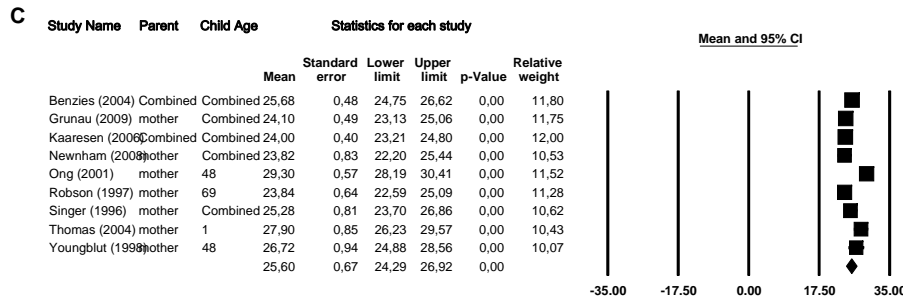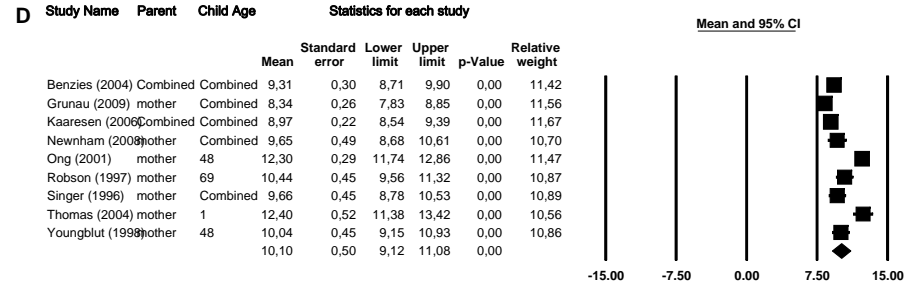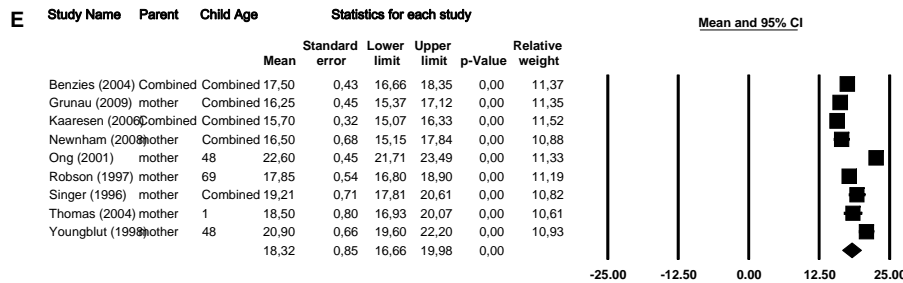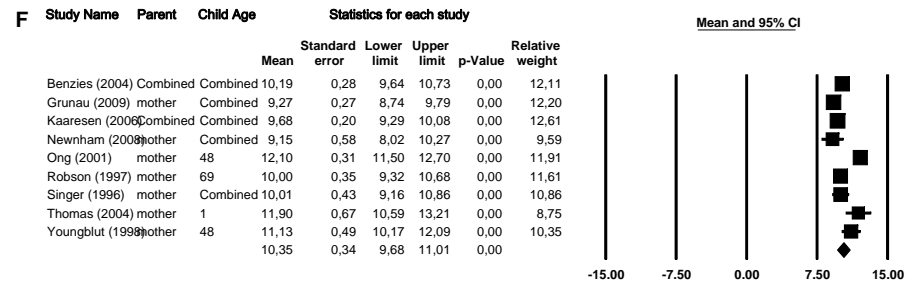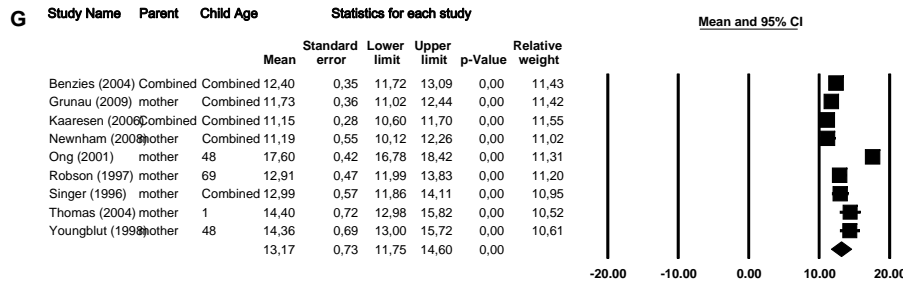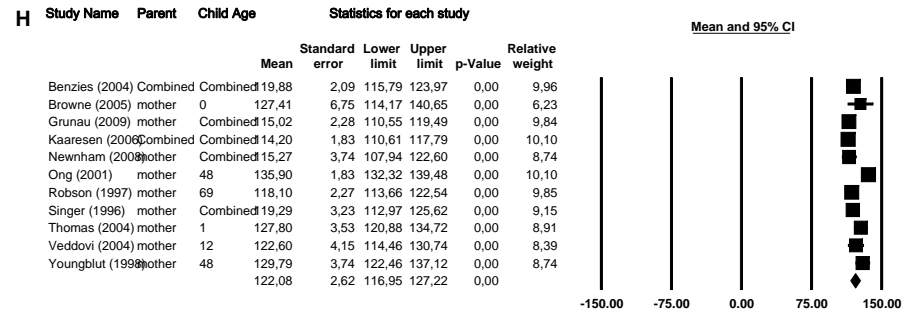

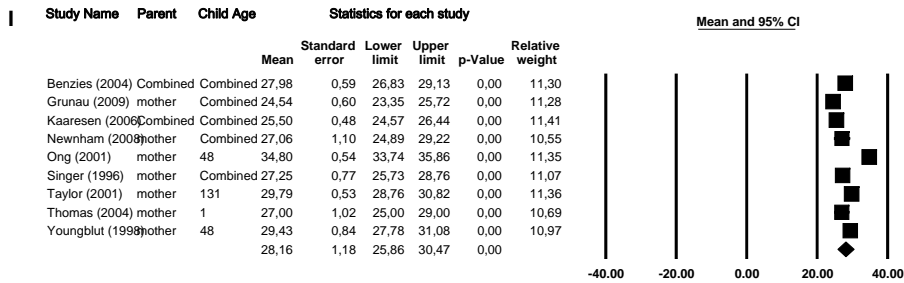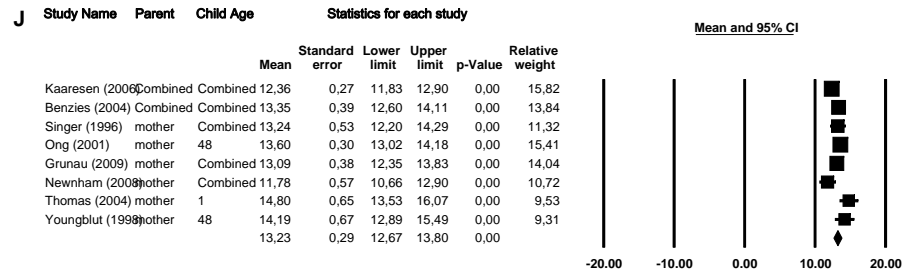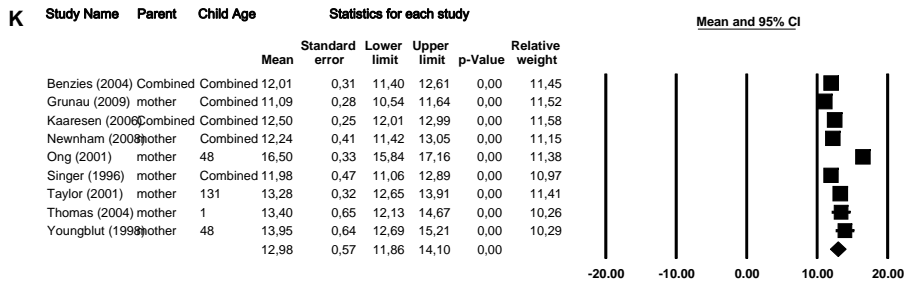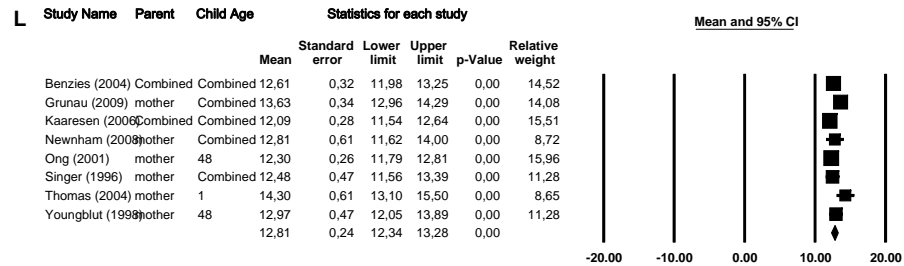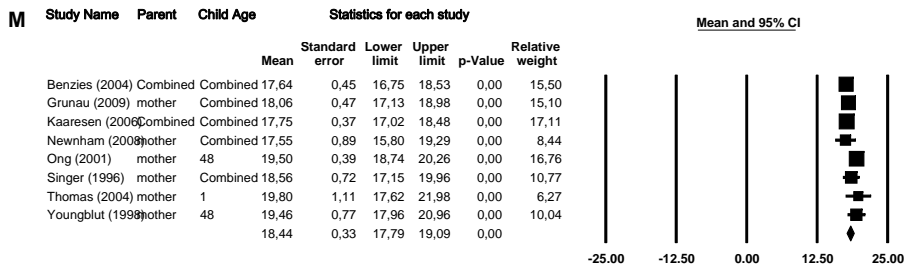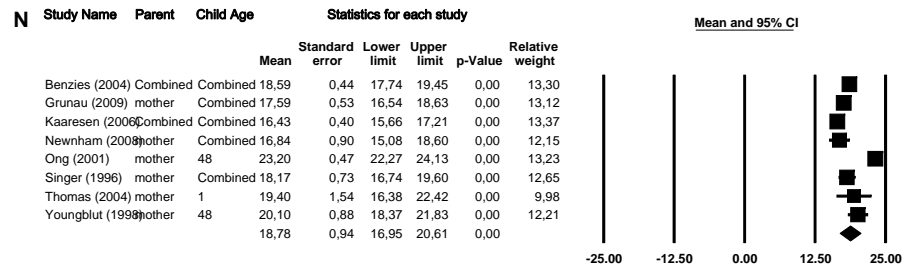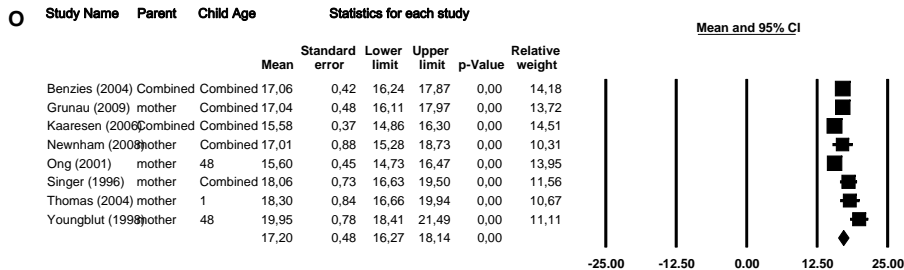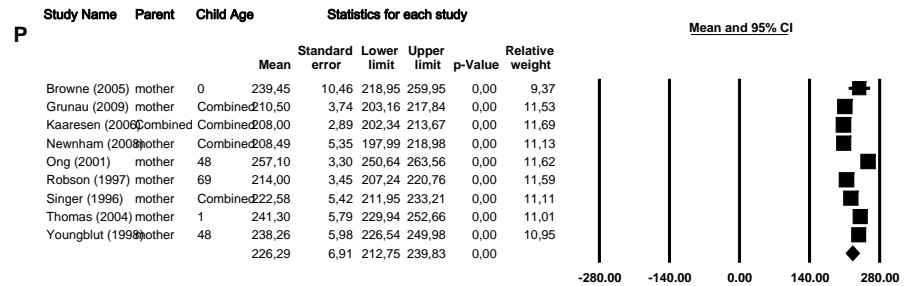

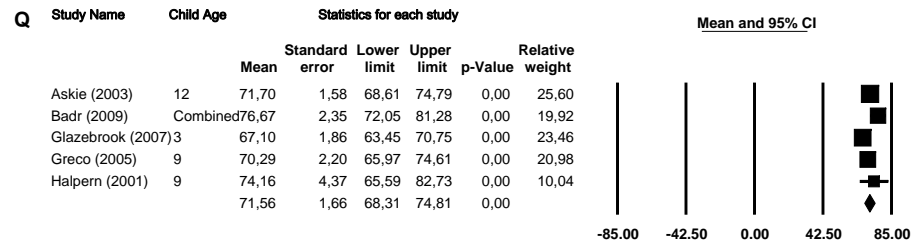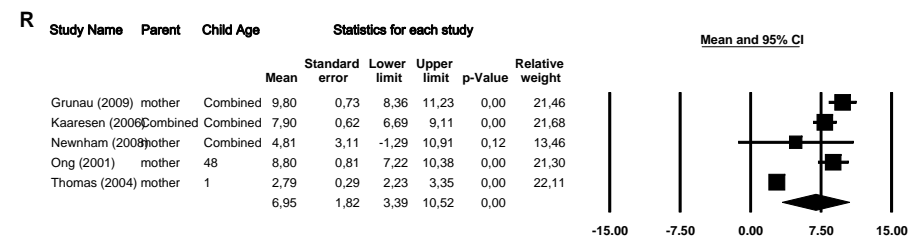

Supplement: Figure S2 — Forest Plots for Meta-Analytic Results of Parents’ Mean PSI Scores. A. Child Domain; B. Distractibility/Hyperactivity; C. Adaptability; D. Reinforces Parent; E. Demandingness; F. Mood; G. Acceptability; H. Parent Domain; I. Competence; J. Isolation; K. Attachment; L. Health; M. Role Restriction; N. Depression; O. Spouse; P. PSI Total; Q. PSI-SF Total; R. Life Stress. (PDF) [file pone.0054992.s002.pdf]

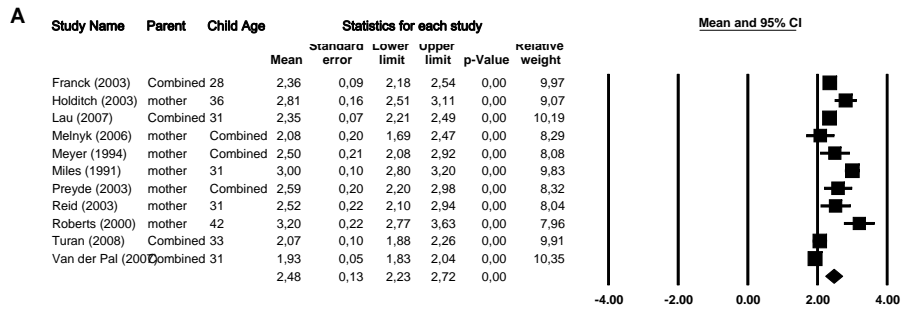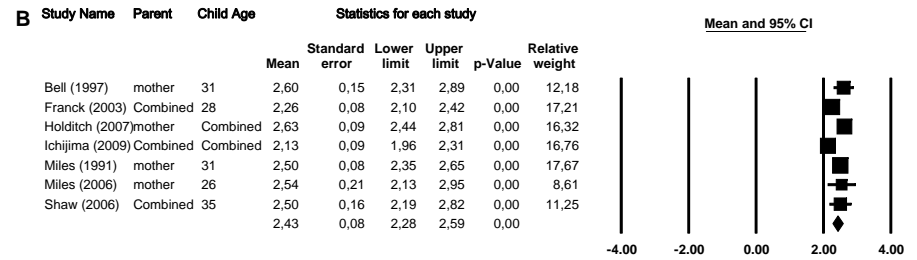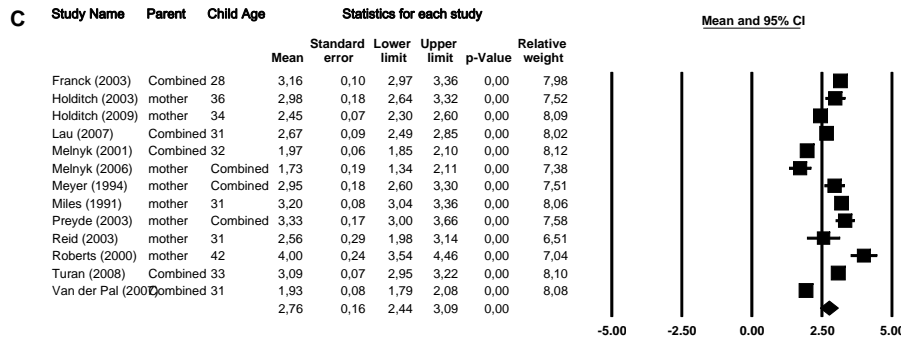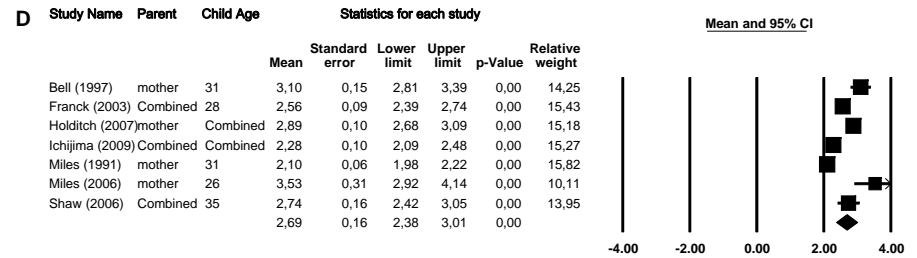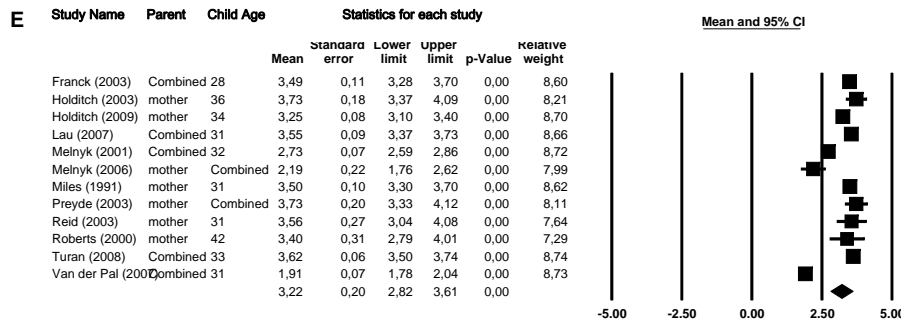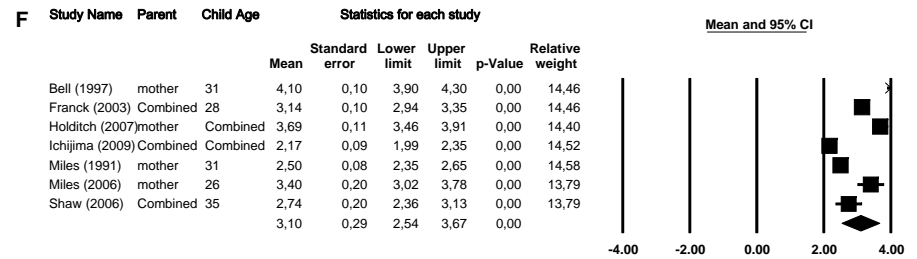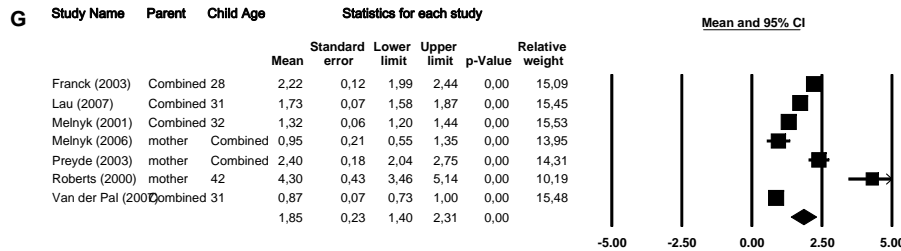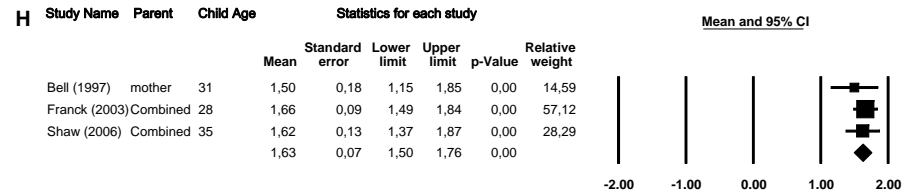

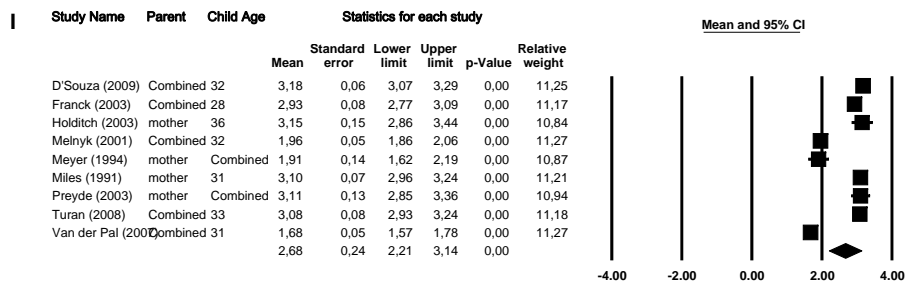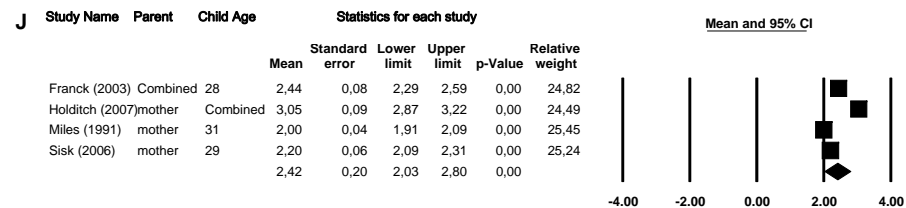

Supplement: Figure S3 — Forest Plots for Meta-Analytic Results of Parents’ Mean PSS:NICU Scores. A. Sights and Sounds Metric 1; B. Sights and Sounds Metric 2; C. Infant Appearance Metric 1; D. Infant Appearance Metric 2; E. Parental Role Alteration Metric 1; F. Parental Role Alteration Metric 2; G. Staff Communication Metric 1; H. Staff Communication Metric 2; I. PSS:NICU Total Metric 1; J. PSS:NICU Total Metric 2. (PDF) [file pone.0054992.s003.pdf]

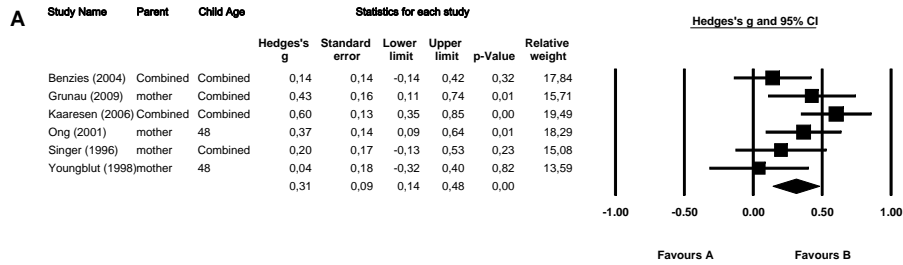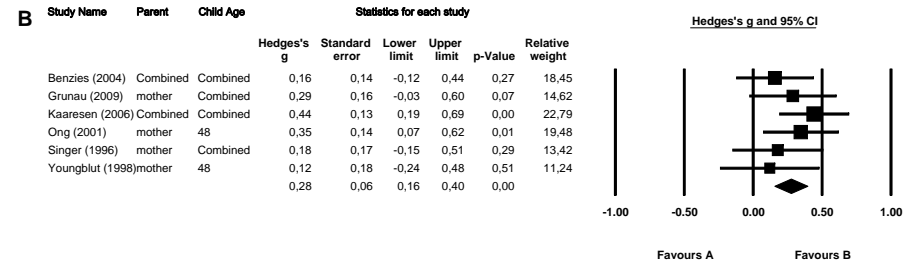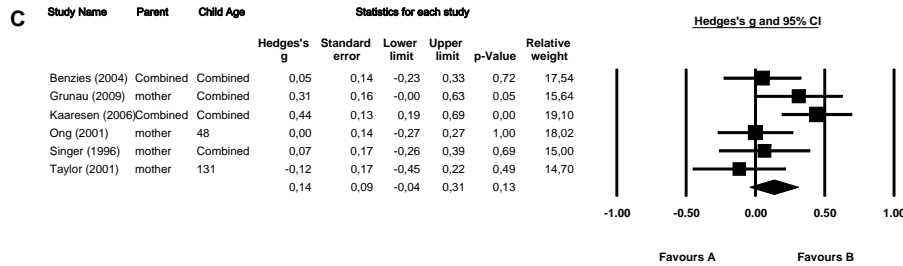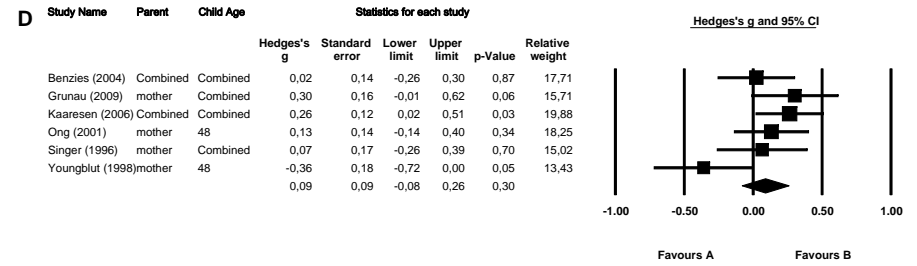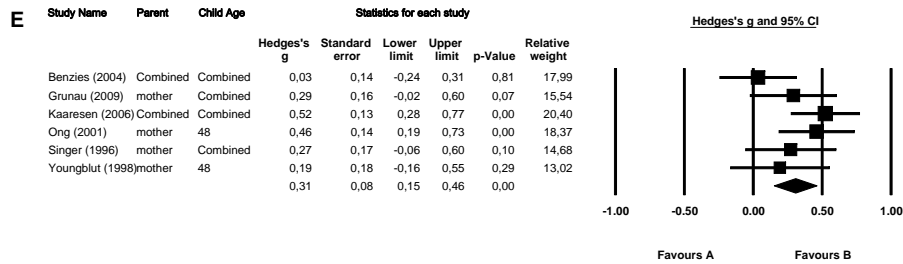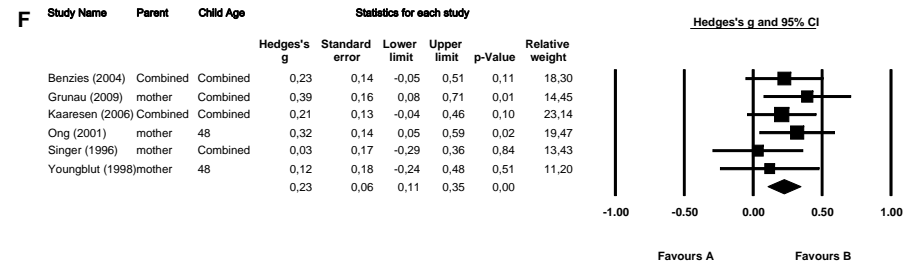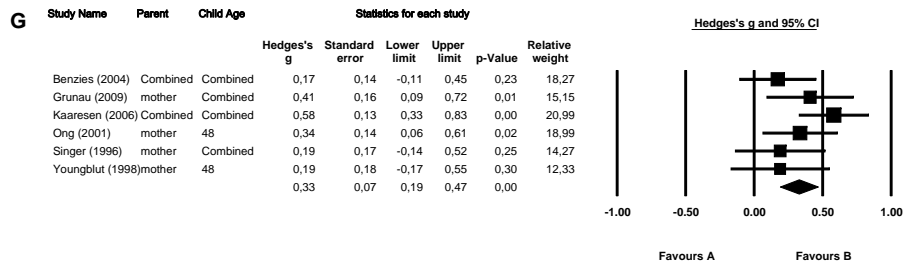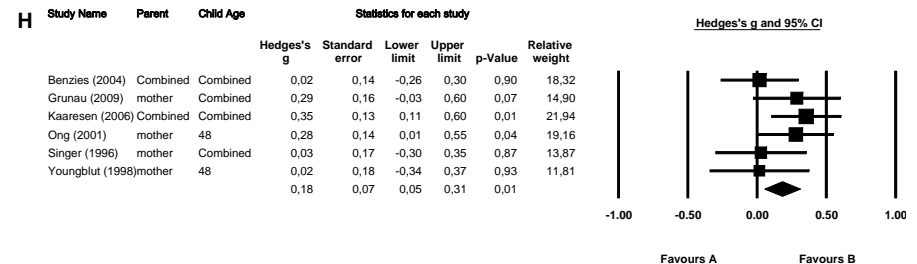

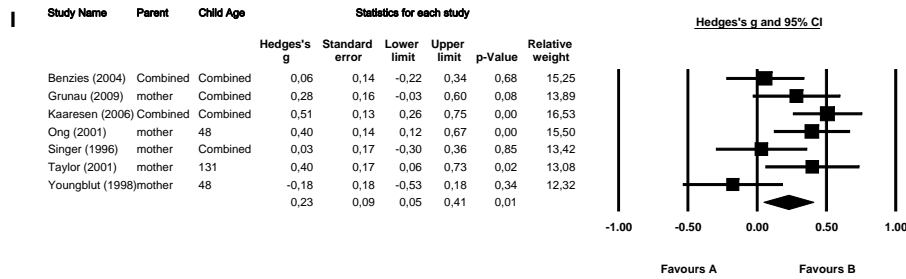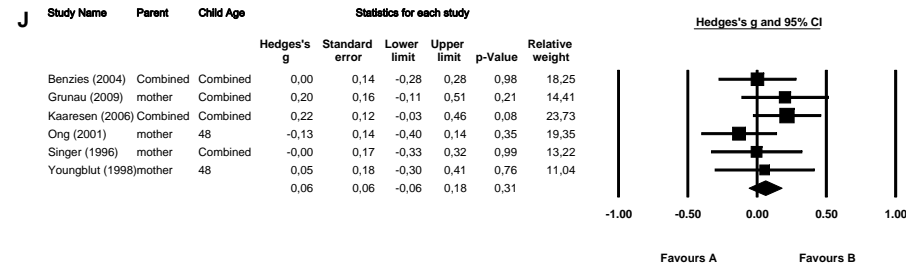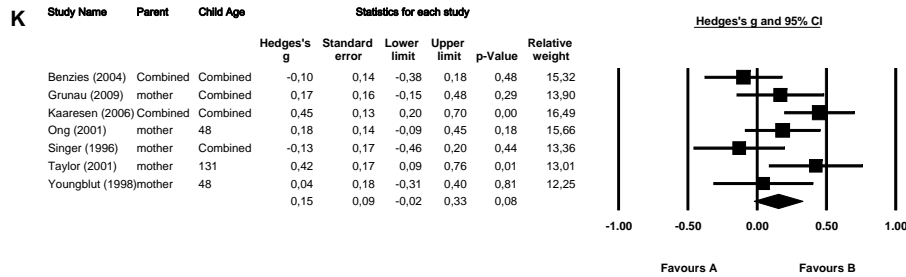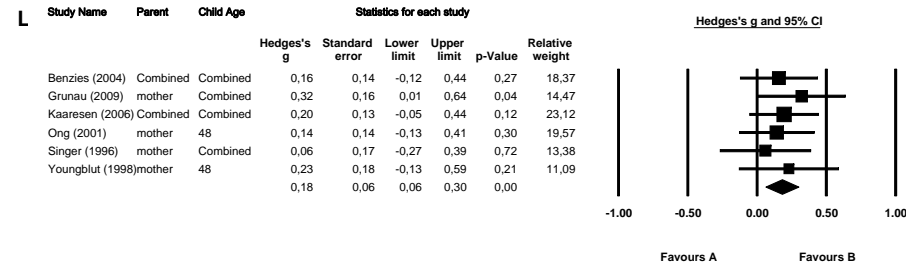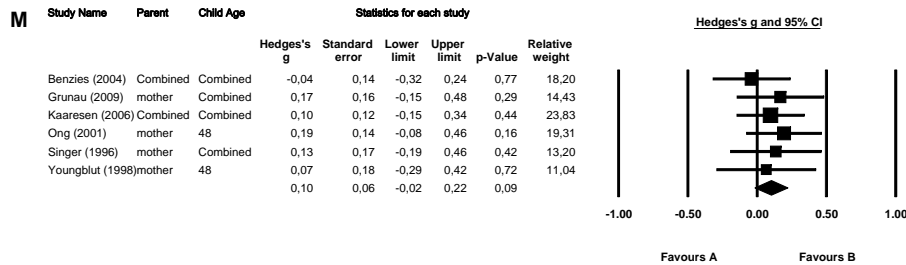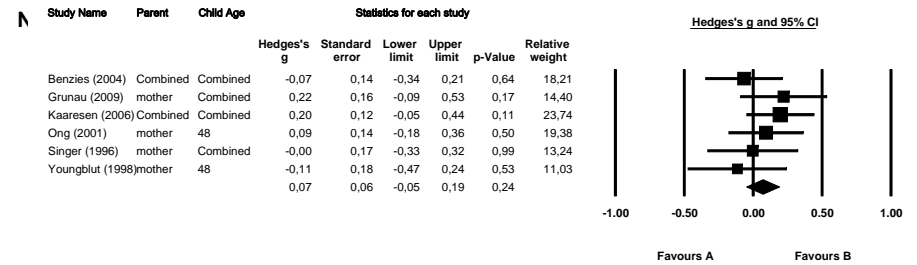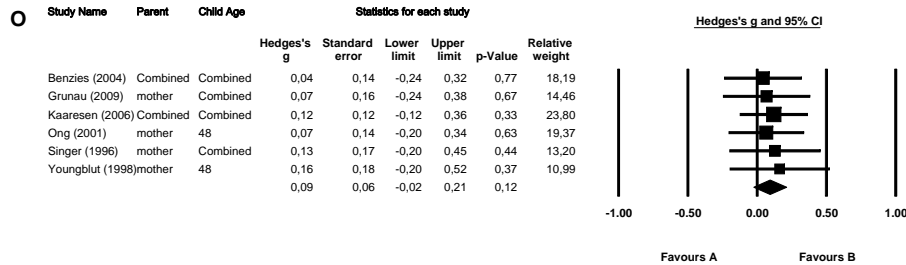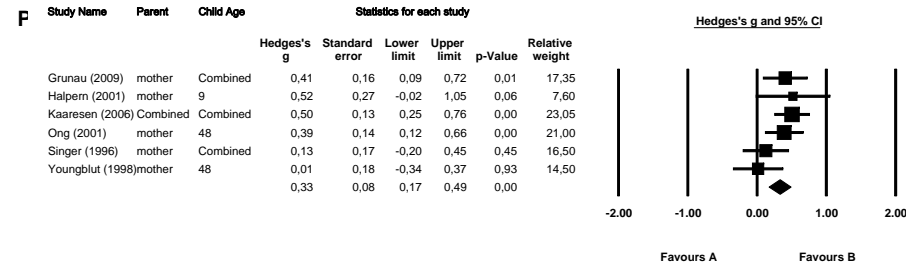

Q

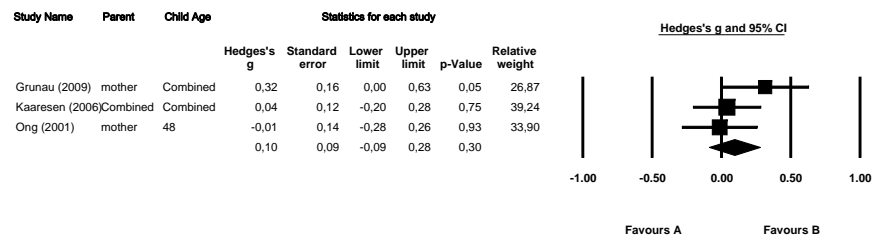

Supplement: Figure S4 — Forest Plots for Meta-Analytic Results of Standardized Differences in PSI Scores Between Parents of Preterm-Born and Term-Born Children. Note. Favours A = Parents of term-born children experience more stress than parents of preterm-born children; Favours B = Parents of preterm-born children experience more stress than parents of term-born children. A. Child Domain; B. Distractibility/Hyperactivity; C. Adaptability; D. Reinforces Parent; E. Demandingness; F. Mood; G. Acceptability; H. Parent Domain; I. Competence; J. Isolation; K. Attachment; L. Health; M. Role Restriction; N. Depression; O. Spouse; P. PSI Total; Q. Life Stress. (PDF) [file pone.0054992.s004.pdf]

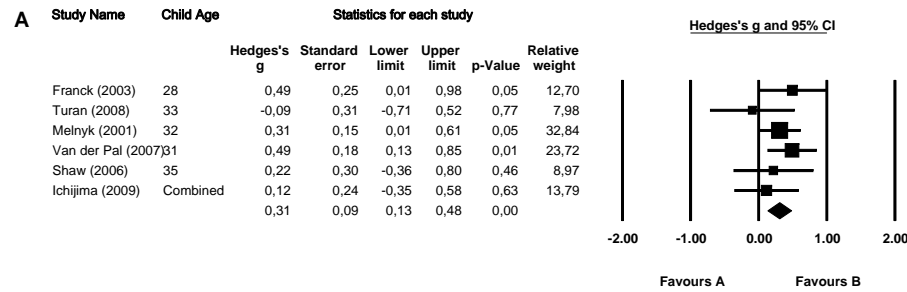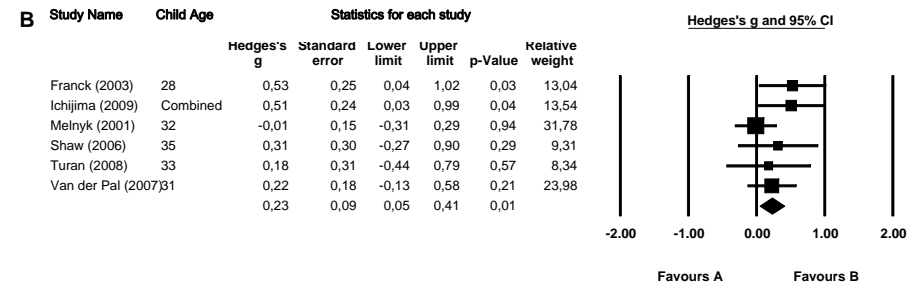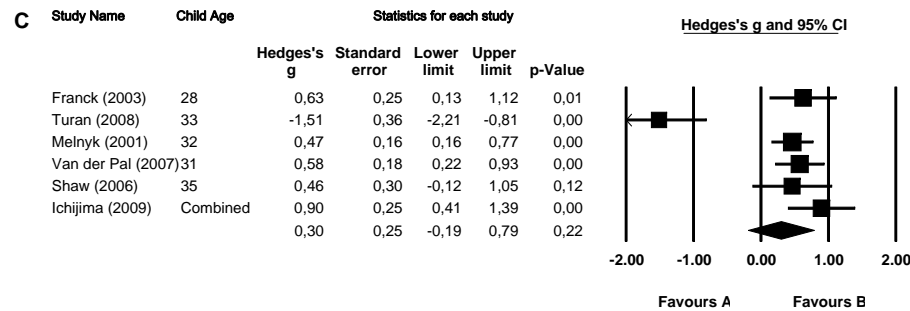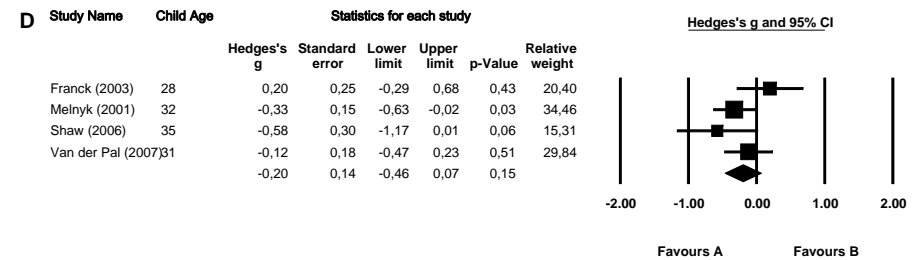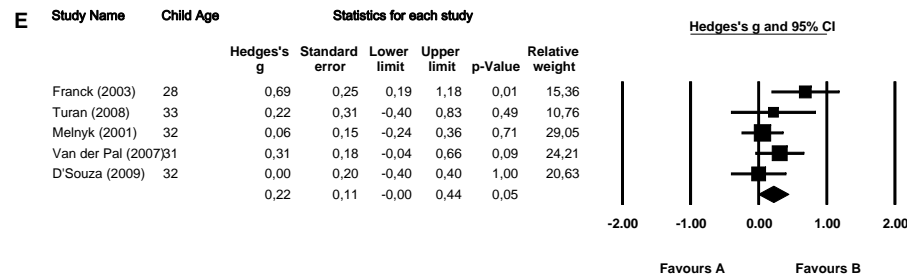

Supplement: Figure S5 — Forest Plots for Meta-Analytic Results of Standardized Differences in PSS:NICU Scores Between Mothers and Fathers of Preterm-Born Children. Note. Favours A = Fathers experience more stress than mothers; Favours B = Mothers experience more stress than fathers. A. Sights and Sounds; B. Infant Appearance; C. Parental Role Alteration; D. Staff Communication; E. PSS:NICU Total. (PDF) [file pone.0054992.s005.pdf]
